# Inflammatory Factors Derived From Metabolic Dysfunction‐Alcoholic Fatty Liver Disease: Inducers of Anxiety and Spatial Memory Impairment

**DOI:** 10.1155/mi/5555342

**Published:** 2026-02-09

**Authors:** Tian-Tian Peng, Yu Shi, Rui Yu, Yu-Xin Nie, Yan Mu, Tong Jin, Jia-Ni Zhang, Xu Wang, Qian Hua, Yan Tan, Ranjitsinh V. Devkar

**Affiliations:** ^1^ School of Life Sciences, Beijing University of Chinese Medicine, Beijing, 100029, China, bucm.edu.cn; ^2^ School of Acupuncture-Moxibustion and Tuina, Beijing University of Chinese Medicine, Beijing, 100029, China, bucm.edu.cn; ^3^ CAS Key Laboratory for Biomedical Effects of Nanomaterials and Nanosafety, Chinese Academy of Sciences and National Center for Nanoscience and Technology of China, Beijing, 100190, China; ^4^ School of Traditional Chinese Medicine, Beijing University of Chinese Medicine, Beijing, 100029, China, bucm.edu.cn

**Keywords:** cognitive decline, liver–brain axis, metabolic dysfunction-associated steatotic liver disease (MASLD), neurodegeneration, neuroinflammation

## Abstract

Metabolic dysfunction‐associated steatotic liver disease (MASLD) has emerged as a global epidemic, with growing evidence suggesting its adverse impact on brain function. However, the underlying mechanisms linking hepatic metabolic dysfunction to neurodegeneration remain unclear. In this study, we systematically investigated the liver–brain axis by integrating genetic epidemiology, experimental neuroscience, and transcriptomics techniques. Two‐sample Mendelian randomization (MR) analysis revealed a potential causal relationship between MASLD and cognitive decline. These findings were validated in a high‐fat diet (HFD)–induced MASLD mouse model, which exhibited hallmark features of metabolic dysfunction, including significant body fat accumulation and elevated serum levels of pro‐inflammatory cytokines (interleukin‐6 [IL‐6] and tumor necrosis factor‐α[TNF‐α]). Behavioral assays demonstrated pronounced anxiety‐like behaviors and impaired spatial memory. Neuropathological analysis revealed neuronal loss and structural alterations in the hippocampal dentate gyrus (DG), accompanied by astrocyte remodeling and M1 microglial polarization, indicating neuroinflammation‐driven disruption of hippocampal circuits. At the molecular level, MASLD altered the expression of key hippocampal genes—including *TCF7L2*, *LCN2*, and *AQP1*—impacting immune response, lipid metabolism, and apoptotic pathways, which collectively contributed to cognitive deficits. Dual immunofluorescence staining, combined with Sholl and 3D analysis quantitatively characterized neuroglial morphological and functional changes, providing structural‐level evidence for MASLD–related brain dysfunction. Taken together, our findings identify MASLD as a modifiable risk factor for neurodegeneration, with systemic inflammation playing a pivotal role in the liver–brain axis. This study highlights key genes and pathways underlying MASLD–induced cognitive impairment, advances understanding of metabolic‐neural cross talk, and offers potential therapeutic targets for mitigating cognitive decline through intervention in the liver–brain axis, developing intervention strategies and highlight the therapeutic promise of targeting the liver–brain axis.

## 1. Introduction

Metabolic dysfunction‐associated fatty liver disease (MASLD) has rapidly become the most prevalent chronic liver disease globally, evolving from a primarily digestive system disorder to a complex systemic metabolic syndrome with wide‐ranging implications for public health [1]. Epidemiological studies reveal that the global prevalence of MASLD in adults exceeds 25%, with an alarming rise in cases among younger populations [2]. Beyond its well‐established hepatic manifestations, MASLD is linked to a host of systemic complications, including insulin resistance and cardiovascular diseases, which pose significant challenges to clinical management.

In recent years, increasing attention has been paid to the relationship between MASLD and central nervous system (CNS) dysfunction, driven by advances in understanding the “gut–liver–brain axis”—a complex regulatory network that connects metabolic processes to brain function [3]. Clinical evidence now clearly demonstrates that patients with MASLD face a markedly higher risk of neurological impairments, including memory decline and cognitive dysfunction, compared to healthy individuals. Some of the most severe cases even show noticeable hippocampal atrophy [4, 5], suggesting that MASLD may play a role in the pathogenesis and progression of neurodegenerative diseases through specific pathological mechanisms.

Research on the neurocognitive impact of MASLD is still in its early stages, particularly regarding the underlying mechanisms. It is well‐established that patients with MASLD exhibit a state of chronic low‐grade inflammation, with the liver acting as a major source of pro‐inflammatory cytokines. These cytokines can cross the blood–brain barrier, potentially influencing CNS function [6]. Moreover, intestinal barrier dysfunction associated with MASLD leads to endotoxin translocation, further amplifying systemic inflammation. Growing evidence suggests that MASLD is not limited to liver dysfunction but is also a key driver of neuroinflammation, contributing to cognitive decline [7]. The precise mechanisms linking hepatic inflammation to neurocognitive impairment, however, remain poorly understood.

While the progression from MASLD to metabolic dysfunction– associated steatohepatitis (MASH) and subsequent liver fibrosis is well‐documented [8], the mechanisms by which MASLD–induced metabolic dysfunction leads to synaptic degeneration and hippocampal damage remain a significant gap in current knowledge. To address this gap, the present study employs an innovative multidimensional approach that integrates Mendelian randomization (MR), neurohistology, and transcriptomic analysis. This strategy aims to systematically investigate the mechanisms by which MASLD induces neurocognitive abnormalities. The central hypothesis is that MASLD–related inflammation directly or indirectly causes neuronal damage, accompanied by functional imbalance in astrocytes and microglia. These changes are thought to alter the expression of key inflammatory genes, such as *TCF7L2*, *Lcn2*, *Ifi204*, *Ifit1*, and *Ifi211* in the hippocampus, ultimately contributing to memory dysfunction.

This multidimensional research framework provides a comprehensive approach to understanding the molecular mechanisms underlying liver–brain communication. The findings will not only enhance our understanding of the systemic impact of MASLD but also provide a theoretical foundation for developing novel therapeutic strategies targeting hepatic inflammatory pathways to mitigate neurodegenerative changes in the CNS.

## 2. Materials and Methods

### 2.1. Animal Model

All animal experiments in this study strictly complied with relevant ethical regulations. The experimental animals were provided by Sipeifu (Beijing) Biology Technology Co. (the company’s animal supply qualification number: 110324241106416058), and all were male mice. Male mice were selected based on previous research reports that female C57BL/6J mice have strong resistance to diet‐induced obesity and insulin resistance, which is not conducive to achieving the purpose of this experiment [9, 10].

The experimental mice were randomly placed in five cages and adaptively raised for 1 week in an environment, where the temperature was precisely controlled at 22 ± 2°C, and the light cycle was strictly set to 12 h of light and 12 h of darkness alternately. During this period, the mice could freely access water. At the same time, the mice were fed with two different kinds of feed for 16 weeks. One of the feeds was a standard chow feed (control, 10% kcal fat, D12450J, Research Diets, New Brunswick, NJ); the other was a high‐fat feed (high‐fat diet [HFD], 40% kcal from fat (palm oil), 20% kcal from fructose, and 2% cholesterol [CHOL]; D09100310, Research Diets, New Brunswick, NJ) to explore the effects of different diets on the mice.

**Figure 1:**
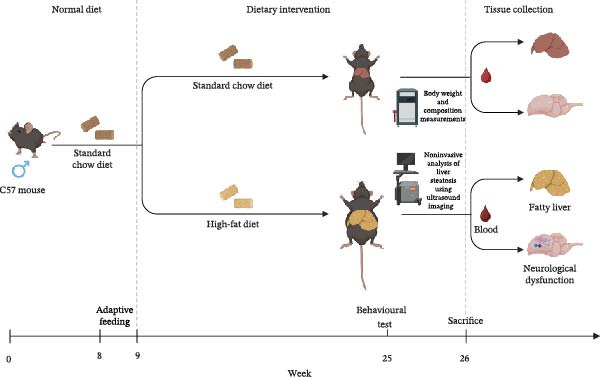
Flowchart of animal experiment. Male C57 mice aged 8 weeks were first subjected to adaptive feeding for 1 week. Subsequently, they were fed with two distinct types of feed for 16 weeks. After that, the mice were euthanized by intraperitoneal injection of 0.5 mL/10 g of avertin. Finally, tissues such as blood, liver, and brain were collected. Created with BioRender.com.

In the experimental operation process, the mice were first anesthetized by intraperitoneal injection of avertin at a dosage of 0.3 mL/10 g to ensure the smooth progress of subsequent operations. When it was necessary to euthanize the mice during the experiment, 0.5 mL/10 g avertin was carefully injected into the abdominal cavity of the mice (Figure 1). All experimental procedures were approved by the Animal Care and Use Committee of the Beijing University of Chinese Medicine (BUCM‐22024070401‐3011), comprehensively ensuring the scientific and compliant nature of the experiment.

### 2.2. Body Weight and Composition Measurements

In this study, the body composition analysis of all mice was precisely carried out using the EchoMRI system (manufactured by LLC, Houston, TX, USA) at the 16th week of their feeding regimen. To minimize the influence of their diet, the mice were required to fast for 4–6 h before the measurement. The device containing the mice was carefully aligned and placed inside the scanner during the actual measurement. This device could effectively restrain the movements of the mice while ensuring unobstructed breathing and physical integrity. The operators then selected the multiecho spin‐echo sequence and finely adjusted the scanning area to comprehensively cover the entire body of the mice, with the resolution calibrated to the range of 0.5–1 mm.

After scanning, the software that came with the EchoMRI system was used to process the collected data, leveraging the signal differences to clearly segment and quantify the fat and lean mass components. At the end of the feeding period, a comparative analysis was performed between different groups to evaluate the percentage changes in fat mass and lean mass. Additionally, the body weight of each mouse was recorded weekly using a digital balance for continuous monitoring.

### 2.3. Behavioral Experiments

#### 2.3.1. Open Field (OF)

OF test to assess anxiety‐like behavior was performed using an arena divided into virtual quadrants. Mice were allowed to become accustomed to a behavioral chamber, including background white noise, for at least 30 min before starting the test. Individual mice were then placed in an OF arena (made of opaque white plastic material, 35 cm × 35 cm) by blind experimenters and allowed to explore for 10 min. The video analysis software was used to quantify the total distance (in centimeters), speed (in millimeters per second), and the proportion of time spent in the central, peripheral, and corner regions, whereas anxiety was evaluated according to the time spent exploring the center zone.

#### 2.3.2. Y‐Maze

A Y‐maze experiment was performed 1 day after the OF experiment to assess cognitive functions. The Y‐maze consists of three arms: the start arm, the familiar arm, and the novel arm. Each arm is 35 cm long, 20 cm high, and 10 cm wide [11, 12]. Each of the arms is connected to the other arms at a 120° angle. Before the start of the experiment, all arms were wiped with 75% alcohol, and a wooden board was placed to close the route into the novel arm. Each mouse was put from the end of the start arm to move freely between the start arm and the familiar arm for 10 min to adapt to the device. After adaptation, the mouse was put back into the cage and allowed to rest for 4 h. Next, wipe all arms with 75% alcohol. The board used to close the access channel into the novel arm was removed so that the mouse had free access to all arms, and then, the mouse was placed at the end of the starting arm. A camera was used to record the movement for 5 min. The number of entries into each arm and the frequency of spontaneous alternation were also recorded. The alternation rate was calculated using the formula [(number of spontaneous alternations)/(total input to the arm−2)] × 100%.

### 2.4. Noninvasive Analysis of Liver Steatosis Using Ultrasound Imaging

We used a high‐frequency small animal ultrasound imaging system (Vevo 2100, LAZR) to noninvasively evaluate hepatic steatosis in mice, while they were on a diet for MASH. Mice were anesthetized with isoflurane and individually placed on a mask that was preheated and continuously fed with a gas anesthetic. After the mice were properly positioned, an initial 3D B‐mode (conventional ultrasound) image was taken to obtain an image of the tissue boundary as the anatomical background. Multiple viewing angles (lateral and sagittal) were used to determine the target location within the liver, and then, the transducer was aligned at this location for imaging of the liver–kidney section for noninvasive measurement of the liver–kidney ratio. Professional image processing software (Vevo 2100, VisualSonics) was employed to analyze liver ultrasound data. During the process, each image was meticulously examined, and the mean gray release values of both the liver and kidneys were measured.

### 2.5. Automatic Biochemical Analyzer to Evaluate Liver Function

After collecting blood samples from the mice, the samples were placed in a centrifuge for centrifugation (4°C, 3000 rpm, 10 min) to isolate serum. Upon completion of the preparation, alanine aminotransferase (ALT), aspartate aminotransferase (AST), alkaline phosphatase (ALP), total bilirubin (TBIL), direct bilirubin (DBIL), albumin (ALB), globulin (GLB), low‐density lipoprotein (LDL), CHOL, and other indicators were detected by automatic biochemical analyzer (Beckman Coulter, DxC700 AU). During the reaction, the instrument monitored the changes of the indicators in the reaction system in real‐time through optical, electrochemical, and other detection technologies, and calculated the content of each liver function index according to the standard curve. Finally, the detection results were analyzed using GraphPad Prism to evaluate the liver function status.

### 2.6. Hematoxylin and Eosin (H&E) Staining

After feeding mice on an HFD, liver samples were collected from HFD mice and wild‐type (WT) mice for histological analysis. Newly collected liver samples were weighed using a balance, and the appearance of each sample was photographed using a digital microscope. The liver was fixed in 4% formalin for 48 h. After dehydration by an ethanol layer, it was permeated with xylene and embedded in paraffin. The sample blocks were cut into 5‐μm‐thick sections and stained with H&E. Finally, the slides were observed under a brightfield microscope (ZEISS). Three H&E slides of mice were taken from each group, and three slides were taken from each liver tissue.

### 2.7. Tissue Preparation and Immunofluorescence Analysis

The experimental mice were anesthetized with 1.25% avertin (at a dosage of 0.3 mL/10 g, intraperitoneally injected). Immediately afterward, cardiac perfusion was carried out with 0.9% normal saline. After the perfusion, fixation was performed with 4% paraformaldehyde (pH 7.4). The mouse brains were then collected and postfixed in 4% paraformaldehyde at 4°C for 12 h and subsequently immersed in phosphate‐buffered saline (PBS) containing 30% sucrose at 4°C for dehydration for 24 h. The brain tissues of the mouse hippocampus were coronally cut with a cryo‐slicer (Leica CM1800; Heidelberg, Germany) to a thickness of 40 μm. The slices were incubated for 30 min at room temperature in a PBS–blocking buffer containing 0.5% Triton X‐100 and 3% bovine serum ALB. The primary antibody was incubated at 4°C overnight for 12 h. The primary antibodies included mouse anti‐NeuN (1:1000, 94403S, CST), rabbit anticleaved caspase3 (1:200, 9661S, CST) and rabbit anti‐GFAP (1:500, 80788S, CST). Then, the secondary antibodies Alexa 488 goat anti‐mouse IgG (1:1000, A21202, Thermo Fisher) and Alexa 594 goat anti‐rabbit IgG (1:1000, A21207, Thermo Fisher) were incubated for 1 h at room temperature. After that, DAPI (1:10000, ab228551, Abcam) was added and incubated for 10 min at room temperature. All images were taken under the FV3000 confocal fluorescence microscope. Each group consisted of three mice, and at least four slices of each brain tissue were taken.

### 2.8. Enzyme‐Linked Immunosorbent Assay (ELISA)

To observe the changes of inflammatory factors in peripheral blood, we measured the levels of Interleukin‐6 (IL‐6, 16193) and tumor necrosis factor‐α (TNF‐α, 21926) using a commercial kit (RayBiotech). During the experimental preparation stage, the ELISA kit was taken out and equilibrated to room temperature. The expiration date and the state of the reagents were checked to ensure their quality. The operation strictly followed the instructions of the kit. When adding samples, the standard substances and samples were accurately measured and pipetted into the corresponding wells, and the temperature and humidity were controlled throughout the incubation process. In terms of quality control, positive control, negative control, and standard curve, samples were set up, and the correlation coefficient (*R* ^2^) of the standard curve was ≥0.99. The detection limits were determined through preliminary experiments on a series of diluted standard substances. The minimum detectable dose of IL‐6 was determined to be 2 pg/mL, and the minimum detectable dose of mouse TNF‐α was determined to be 60 pg/mL. In terms of reproducibility, repeated detections were carried out by different personnel at different time points, and the average coefficient of variation (CV) was less than 10%, ensuring reliable results. After the values were read by the microplate reader, the concentration was calculated according to the standard curve.

### 2.9. Cell Culture and IL‐6/TNF‐α Treatment

The HT‐22 cell line (Number CL‐0697, kindly provided by Wuhan Pricella Biotechnology Co., Ltd., on February 15, 2025) is derived from the hippocampal tissue (in situ; Brain, hippocampus; UBERON ID: UBERON_0002421) of *Mus musculus* (Mouse, NCBI Taxonomy: 10090). Its Resource Identification Initiative ID (RRID) is CVCL_0321, with detailed information available on the website: https://www.cellosaurus.org/CVCL_0321. This cell line has been authenticated by STR profiling, yielding a 96.77% match with the ExPASy database and 18 matched loci. The authentication report is included in Supporting Information 1. It has not been reported as misidentified or contaminated and is not listed among problematic cell lines. The cell line is cultured in Hyclone Dulbecco’s modified eagle medium (DMEM)/high glucose (Thermo Fisher Scientific), supplemented with 10% fetal bovine serum (Thermo Fisher Scientific) and 1% penicillin/streptomycin (Thermo Fisher Scientific). This cell line maintained a good state during the culture process and was free of bacterial and mycoplasma contamination. Recombinant mouse IL‐6 and TNF‐α were purchased from R&D Systems (Minneapolis, MN, USA) and reconstituted in PBS. They were diluted with FBS‐free Hyclone DMEM/high glucose to obtain a concentration of 1000 pg/mL. The medium in each well was gently aspirated, and then, IL‐6 or TNF‐α at a concentration of 1000 pg/mL was added for 24 h.

### 2.10. TUNEL and Mito Tracker Green FM Staining Assays

Approximately 100,000 HT‐22 cells were seeded into confocal dishes. The HT‐22 cells were exposed to 1000 pg/mL of IL‐6 or TNF‐α. After 24 h of exposure, the cells were washed three times with PBS 1X. TUNEL (Lab leader) and Mito Tracker Green FM (Invitrogen) staining assays were carried out according to the manufacturer’s instructions. A Nikon AX confocal microscope was used for cell imaging.

### 2.11. Imaging Analysis

Semi‐automatic analysis of images at a 10x magnification was performed using the Image J software to evaluate the area percentage of Neun and GFAP‐positive cells. Through relevant functions of the software, the images were processed and analyzed to calculate the corresponding area percentage. For assessing the cell numbers and calculating the average width of dentate gyrus (DG), coronal sections showing dorsal (Bregma −1.91 mm to −2.27 mm) were chosen. On the high‐resolution tissue section images, we manually marked the starting and ending points along the vertical direction of DG. The software then measured the distance between these two points in pixel units. To guarantee accuracy, we conducted 6–10 measurements on the DG region of each sample and subsequently calculated the average value, which was taken as the width of DG for that particular sample. After computing the average width of the DG for all samples, it was utilized for subsequent data analysis [13].

For the confocal stacks obtained at an 80x magnification, reconstruction was carried out in 3D mode using the Imaris software. During this process, based on the morphological characteristics of GFAP‐positive cells and IBA‐positive cells, they were reconstructed in three‐dimensional space to observe their stereoscopic structures better. Meanwhile, the Imaris software was used to evaluate the cell body volume and the number of branches of individual cells, which helped to gain a more detailed understanding of the structural details and physiological characteristics of cells and provided more quantitative information.

### 2.12. MR Analyses to Assess Causality

This study design adhered to the guidelines outlined in Strengthening the Reporting of Observational Studies in Epidemiology using MR (STROBE‐MR; Supporting Information 2: Table S1). The characteristics of the GWAS data sources used in this study are summarized in Supporting Information 2: Table S2. We conducted a two‐sample MR analysis to investigate the causal relationship between MASLD and cognitive functions. Effect estimates were obtained using the inverse variance weighting (IVW) method, a traditional MR analysis approach. All main analysis methods and sensitivity tests were conducted using the “TwoSampleMR,” “tidyverse,” and “ggplot2” packages in R software version 4.3.2. These packages provided the necessary functions for performing MR analyses and for data visualization.

### 2.13. mRNA Sequencing

Total RNA was extracted from tissues using TRIzol (Invitrogen, Carlsbad, California, USA) according to the manual instructions. Library preparation, mRNA sequencing and initial analyses were performed by BGI (China). The sequencing data were subjected to quality control prior to further analyses. Sequencing was conducted on the DNBSEQ platform with a paired‐end read length of 150 bp (PE150). The raw sequencing data were filtered using SOAPnuke software (v1.5.6) to obtain clean data. Clean reads were aligned to the reference gene set using Bowtie2 (v2.3.4.3). Gene expression quantification was performed with RSEM software (v1.3.1), and a clustered heatmap of gene expression levels across different samples was generated using the pheatmap package (v1.0.8). Differential gene detection was carried out using DESeq2 (v1.4.5; or DEGseq/PoissonDis) with the criteria of *Q* value ≤0.05 or FDR ≤0.001. For further exploration of gene functions associated with phenotypic changes, Gene Ontology (GO; http://www.geneontology.org/) and Kyoto Encyclopedia of Genes and Genomes (KEGG; https://www.kegg.jp/) enrichment analyses of differentially expressed genes were performed using the phyper function based on the hypergeometric test. Terms with a *Q* value ≤0.05 were considered significantly enriched in the candidate genes. All figures were generated using R software (v4.5.1) and the MicroBioInfo platform.

### 2.14. Real‐Time PCR

Total RNA was extracted using the SteadyPure Universal RNA Extraction Kit (AG21017). For each reverse transcription (RT) reaction, 100 ng of total RNA was used, and cDNA synthesis was performed with M‐MLV reverse transcriptase (AG11706) and random primers. Real‐time polymerase chain reaction was carried out using the QuantStudio 7 Real‐Time PCR System (Applied Biosystems) and SYBR green PrecisionPLUS reagent (PPLUS‐LR, PrimerDesign). All reactions were run in duplicate. The expression level of each sample was normalized to GAPDH as an internal reference and expressed as a percentage relative to the control group. All primers used for quantitative qRT‐PCR were synthesized by Shanghai Sangon Biotech Co., Ltd. The specific primer information is as follows: Tcf7l2 primers: The forward primer (mTcf7l2‐F) sequence is 5′‐CTCACGCCTCTCATCACGTA‐3′, and the reverse primer (mTcf7l2‐R) sequence is 5′‐TCCTGTCGT GATTGGGTACA‐3′; CLDN 1 primers: the forward primer (CLDN 1‐F) sequence is 5′‐GACACCTCCCAGAAGGCAGA‐3′, and the reverse primer (CLDN 1‐R) sequence is 5′‐CGACATTAGTGGCCACAGCA‐3′; AQP1 primers: the forward primer (AQP1‐F) sequence is 5′‐GTCCAAGGGCCACAGACAAG‐3′, and the reverse primer (AQP1‐R) sequence is 5′‐TTGGCACTCTGCAGCTGGTA‐3′; Lcn2 primers: the forward primer (Lcn2‐F) sequence is 5′‐CTGAATGGGTGGTGAGTGTG‐3′, and the reverse primer (Lcn2‐R) sequence is 5′‐GCTCTCTGGCAACAGGAAAG‐3′; internal reference gene GAPDH primers: the forward primer (GAPDH‐F) sequence is 5′‐GAGAGTGTTTCCTCGTCCCG‐3′, and the reverse primer (GAPDH‐R) sequence is 5′‐ATGAAGGGGTCGTTGATGGC‐3′.

### 2.15. Statistical Analysis

All data were presented as mean ± SEM. The statistical analysis was carried out using GraphPad Prism version 8.0.2 statistical analysis software. The comparison between the two groups was statistically tested using the unpaired two‐tailed Student’s test; *p* < 0.05 was statistically significant.

## 3. Results

### 3.1. MR Identifies MASLD as a Causal Risk Factor for Cognitive Impairment and Anxiety

To investigate whether MASLD contributes causally to cognitive impairment, we performed a two‐sample MR analysis using 21 single‐nucleotide polymorphisms (SNPs) extracted from the Finnish R12 cohort (https://www.finngen.fi/en). SNPs that were palindromes or that failed sensitivity testing via the leave‐one‐out procedure were removed to maintain instrument reliability (Supporting Information 2: Table S3). All selected SNPs demonstrated *F*‐statistic greater than 10, indicating sufficient instrument strength for MR and minimizing the likelihood of weak‐instrument bias [14].

Using the IVW method—considered the primary MR analytical approach because it provides the most precise causal estimates under standard assumptions—we identified significant associations between genetically predicted MASLD and multiple cognitive domains. Specifically, higher MASLD liability was causally linked to reduced cognitive processing accuracy (OR = 0.996, 95% CI: 0.992–0.999; *p* = 0.027), poorer nonword reading performance (OR = 0.958, 95% CI: 0.926–0.992; *p* = 0.015), decreased spelling ability (OR = 0.966, 95% CI: 0.934–0.999; *p* = 0.045), and lower phoneme awareness (OR = 0.944, 95% CI: 0.898–0.992; *p* = 0.022). Scatter plots and the full analysis corresponding to these findings are presented in Supporting Information 2: Table S4 and Supporting Information 1: Figure S1. These cognitive domains collectively reflect fundamental components of language processing and executive functioning.

In addition to cognitive deficits, MASLD also showed a significant causal effect on emotional health. Individuals with higher genetic susceptibility to MASLD exhibited an elevated risk of worry and anxiety‐related traits (OR = 1.015, 95% CI: 1.002–1.028; *p* = 0.022; Figure 2).

Figure 2MR analysis linking MASLD to cognitive decline and anxiety‐related traits. (A) Schematic overview of the MR study design. (B) Forest plots illustrating the causal effects of MASLD on cognitive traits and anxiety‐related outcomes. Results were presented as odds ratios (ORs) with corresponding 95% confidence intervals (CIs). Statistical significance was defined as 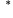 *p* < 0.05.

**Figure:**
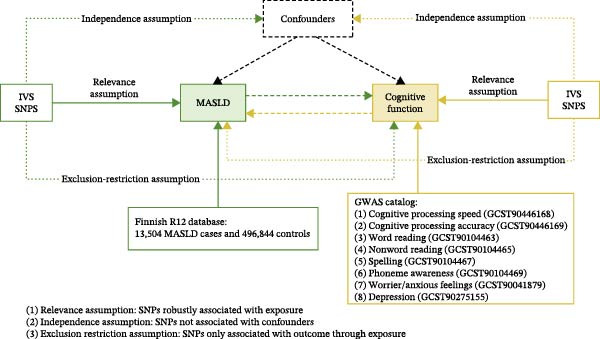
(A)

**Figure:**
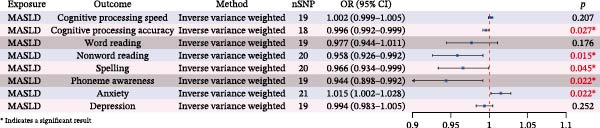
(B)

In all MR analyses, Cochran’s *Q*‐test was employed to assess heterogeneity in the IVW and MR‐Egger regression results, revealing no significant heterogeneity (Supporting Information 2: Table S5). The single‐SNP plots, funnel plots and leave‐one‐out forest plots are displayed in Supporting Information 1: Figure S2–S4. These sensitivity analyses further corroborate the robustness of the primary MR analysis results.

Together, these findings support a causal relationship between MASLD and both cognitive decline and anxiety‐related behaviors, highlighting MASLD as a potential modifiable risk factor for neuropsychological dysfunction.

### 3.2. HFD Induces Metabolic Dysfunction and Hepatic Injury in the MASLD Model Mice

Given that MR analysis revealed a potential causal relationship between MASLD and cognitive dysfunction, it was essential to validate these genetic findings in an experimental setting. We established a HFD–induced MASLD mouse model to recapitulate the metabolic and hepatic abnormalities observed in humans and to provide a platform for subsequent neurobiological analyses.

After 16 weeks of HFD feeding, mice exhibited pronounced metabolic disturbances accompanied by notable phenotypic alterations, including body size and visible vibrissae (whisker) loss (Figure 3A). Body composition analysis demonstrated a 36% increase in overall body weight and a ninefold increase in total fat mass, whereas lean mass and spleen weight remained unchanged compared to controls (Figure 3B–E). These results indicate that HFD feeding successfully induced obesity‐driven metabolic dysfunction without generalized organ enlargement.

Figure 3HFD promotes obesity, increased adiposity, and liver dysfunction in the MASLD model mice. (A) Mouse appearance. (B) Mouse body weight chart. (C) Statistical map of mouse spleen weight. (*n* = 8–10 per group) (D) Percentage of fat mass and (E) lean mass assessed in C57BL6 mice by EchoMRI at 16 weeks of either NC or HFD diet. (F–I) Automatic biochemical analyzer results of the serum levels of ALT, AST, LDL, and CHOL (*n* = 8–10/3–4 per group). The arrow indicates the changes in the vibrissae of the mice. Data are presented as means ± SD.  ^∗^ *p* < 0.05,  ^∗∗^ *p* < 0.01,  ^∗∗∗^ *p* < 0.001 vs. control. Two‐tailed and unpaired Student’s *t*‐test for all data.

**Figure:**
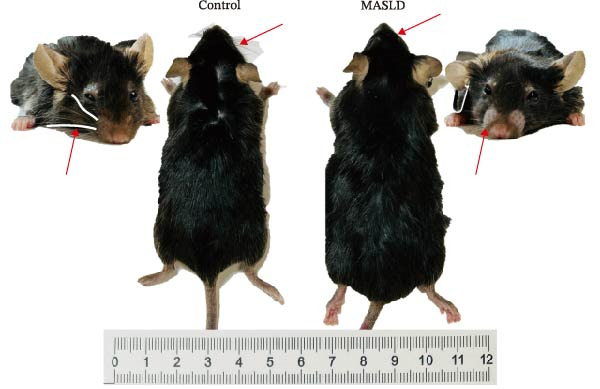
(A)

**Figure:**
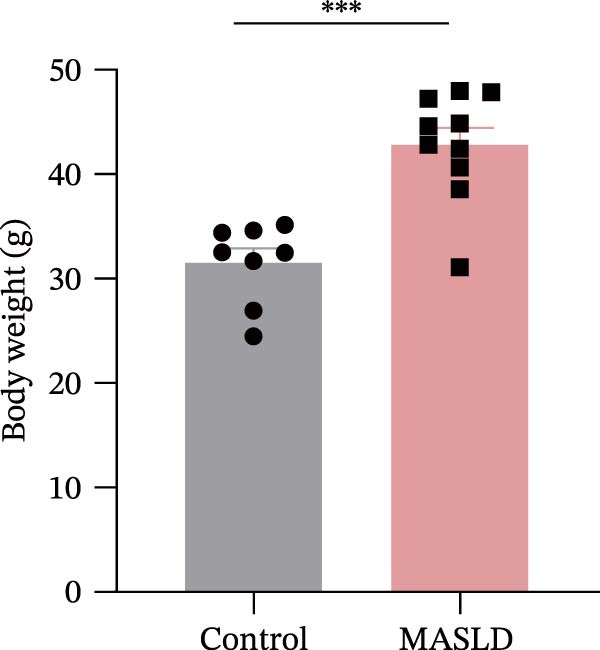
(B)

**Figure:**
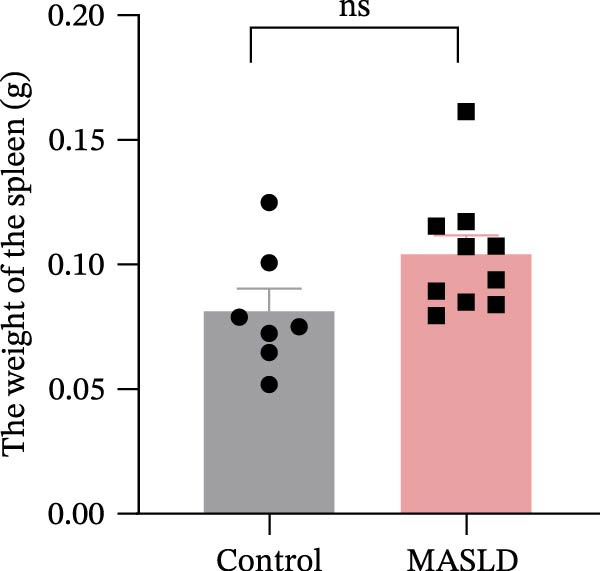
(C)

**Figure:**
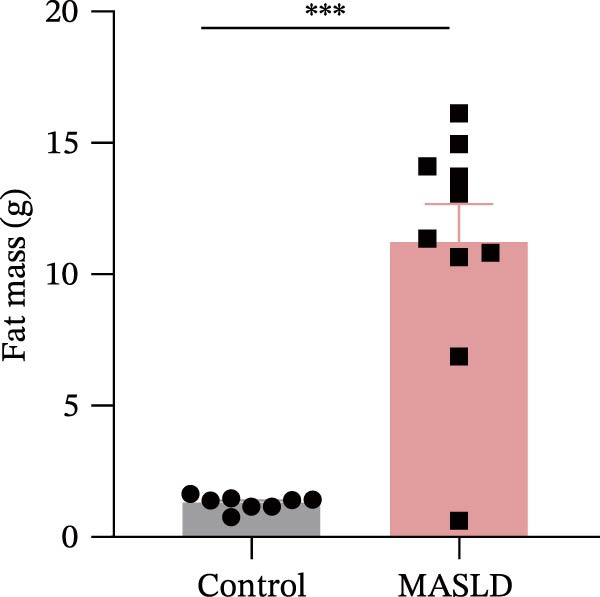
(D)

**Figure:**
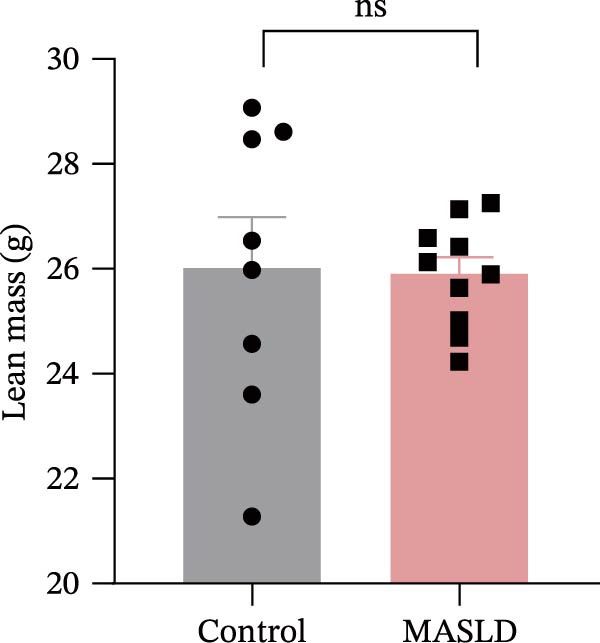
(E)

**Figure:**
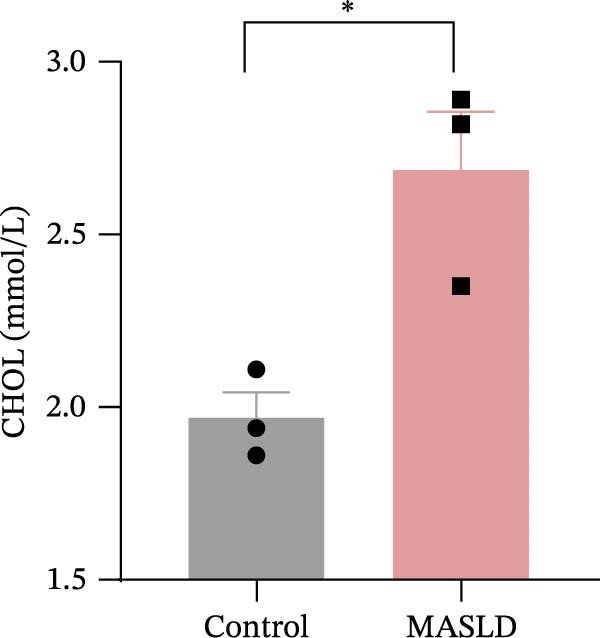
(F)

**Figure:**
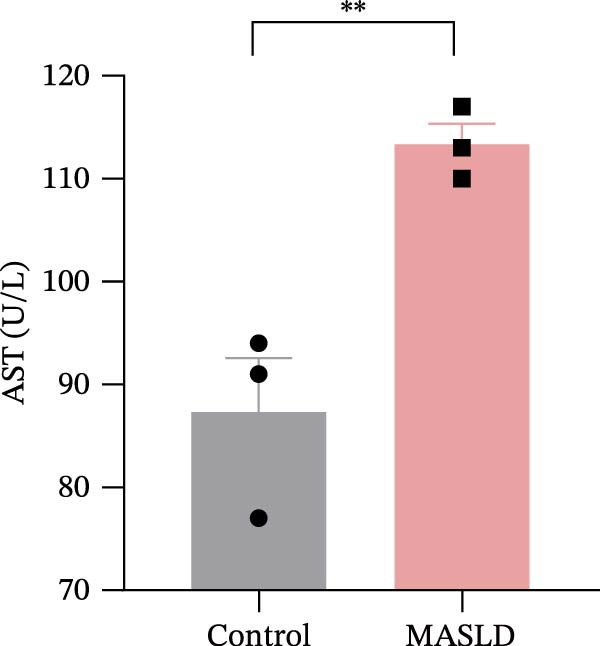
(G)

**Figure:**
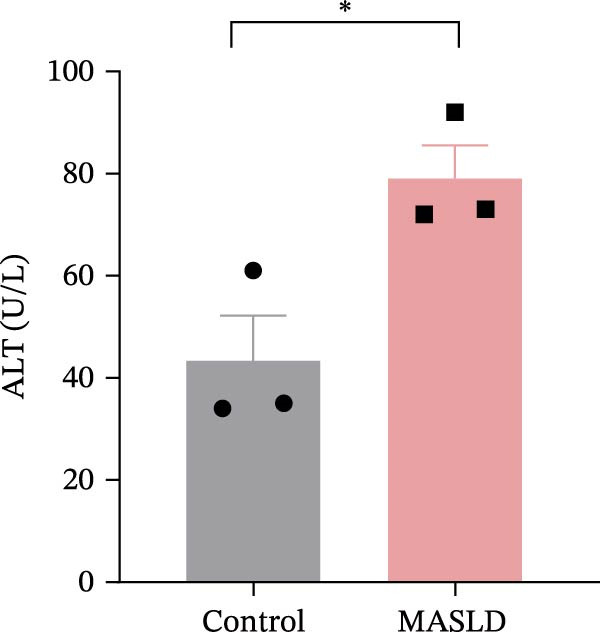
(H)

**Figure:**
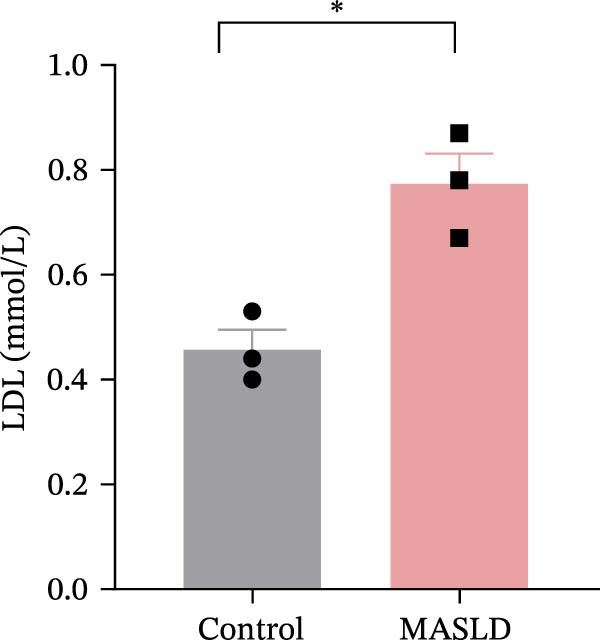
(I)

Consistent with MASLD progression, HFD‐fed mice also developed significant hepatic injury. Serum levels of ALT (+83%) and AST (+30%) were markedly elevated (Figure 3F, G), reflecting hepatocellular damage [15]. In addition, dyslipidemia was evident, with increased total CHOL (+37%) and LDL (+67%) concentrations (Figure 3H, I), indicating impaired hepatic lipid metabolism and clearance [16].

Together, these metabolic, phenotypic, and biochemical changes confirm the successful establishment of a MASLD mouse model, providing a robust in vivo system to investigate downstream neuroinflammatory and cognitive consequences.

### 3.3. HFD Induces Hepatic Steatosis, Systemic Inflammation, and Intestinal Barrier Damage in the MASLD Model Mice

Following confirmation of metabolic dysfunction in HFD‐fed mice, we next assessed key pathological hallmarks of MASLD to ensure the robustness of the model for subsequent neurobiological analyses. Prolonged HFD feeding produced substantial hepatic changes, beginning with a 56% increase in absolute liver weight compared to controls (Figure 4A–D), consistent with excessive hepatic lipid accumulation. This was supported by in vivo ultrasound imaging, which revealed a 2.3‐fold elevation in the liver–kidney grayscale ratio (Figure 4B), indicating enhanced echogenicity characteristic of steatotic liver tissue.

Figure 4HFD induces hepatic steatosis, hepatocellular ballooning, pro‐inflammatory cytokine upregulation, and intestinal tight junction disruption in MASLD mice. (A) Representative ventral and dorsal liver images. (B) Representative ultrasound images of the liver and kidney. The red circle represents the liver, and the blue circle represents the kidney. (C) Liver H&E staining showing diffuse macrovesicular steatosis, lipid droplets, and ballooned hepatocytes. The upper image shows a 100x field of view (scale bar = 200 μm), and the lower image is a 400x magnification of the gray‐boxed area in the upper image (scale bar = 50 μm). Numerous white circular vacuoles (intracellular lipid droplets in hepatocytes) are visible, presenting diffuse macrovesicular steatosis; some hepatocytes exhibit ballooning degeneration (cell swelling with loose cytoplasm, indicated by gray arrows), and red arrows point to lipid droplets and damaged hepatocytes. (D) Liver weight quantification. (E) Liver–kidney grayscale ratios. (F–K) ELISA analysis of IL‐6 and TNF‐α levels in serum, brain, and liver (*n* = 7–10/3–6 per group). (L) Representative immunofluorescence images of colonic tissue showing CD31, ZO‐1, and Occludin staining. From left to right are 10x merged image, magnified image, CD31 (red) single‐staining image, ZO‐1 (green) single‐staining image, and Occludin single‐staining image. (M–O) Quantification of fluorescence intensities (*n* = 3–4 per group). Data are presented as means ± SD.  ^∗^ *p* < 0.05,  ^∗∗^ *p* < 0.01,  ^∗∗∗^ *p* < 0.001 vs. control. Two‐tailed and unpaired Student’s *t*‐test for all data.

**Figure:**
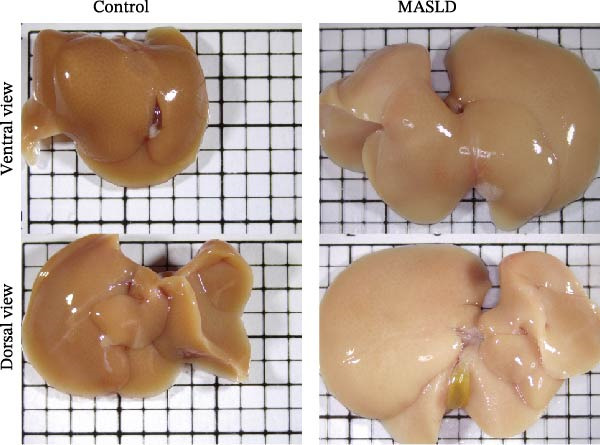
(A)

**Figure:**
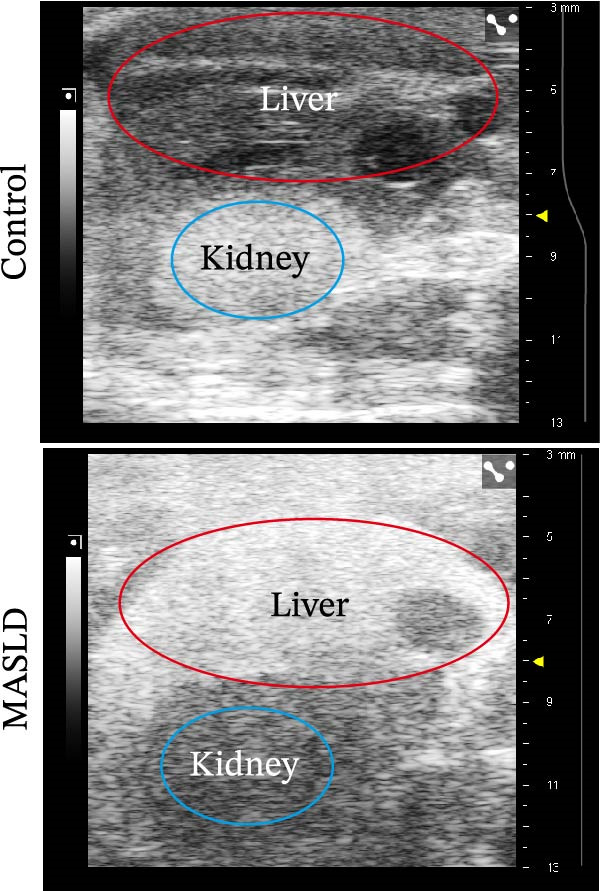
(B)

**Figure:**
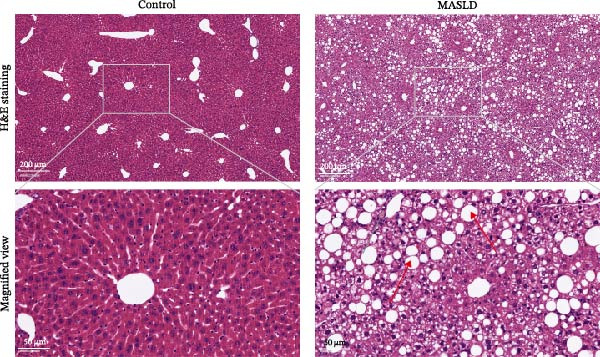
(C)

**Figure:**
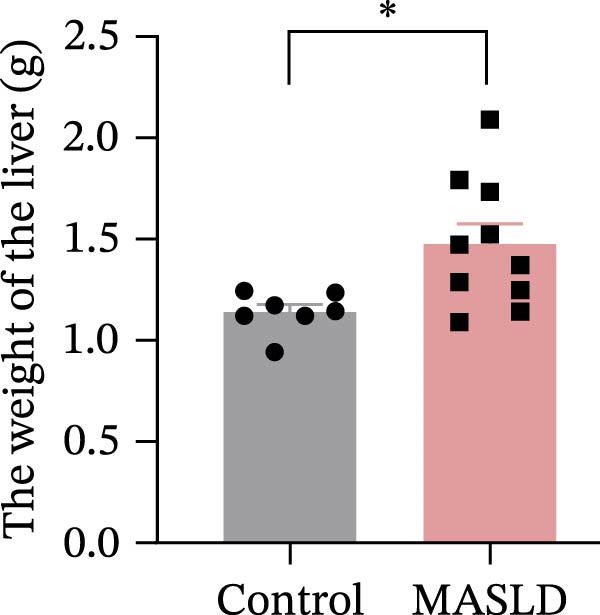
(D)

**Figure:**
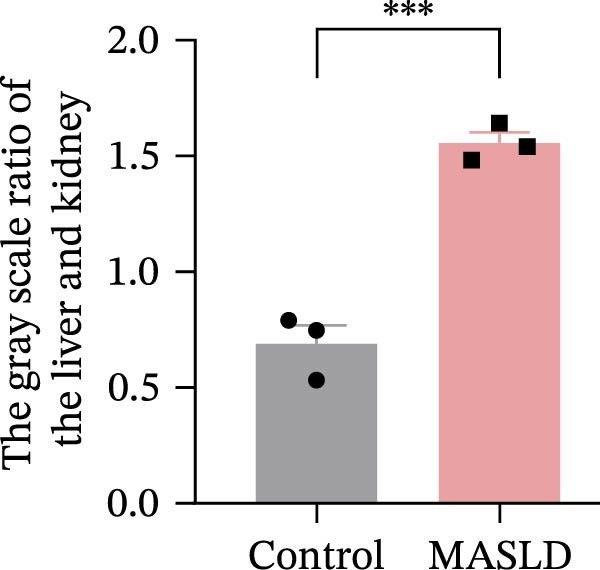
(E)

**Figure:**
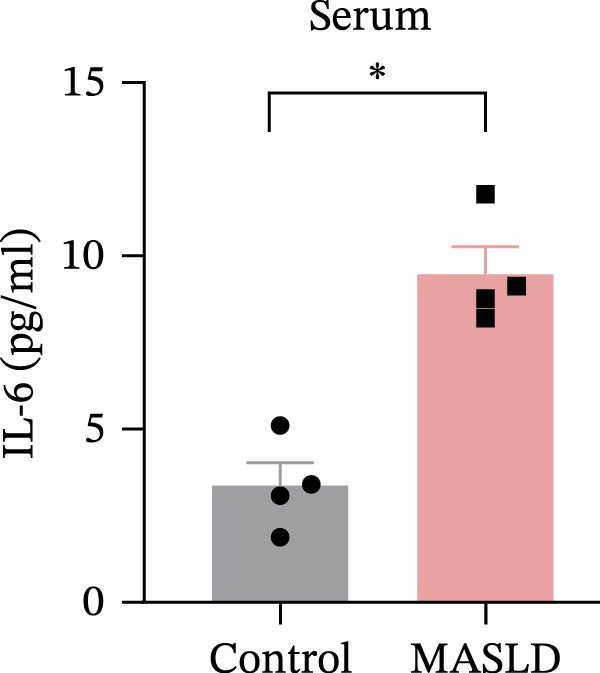
(F)

**Figure:**
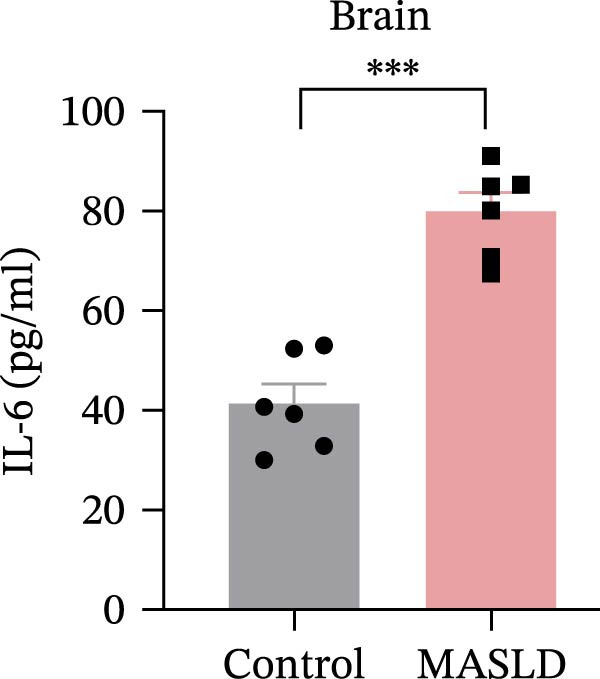
(G)

**Figure:**
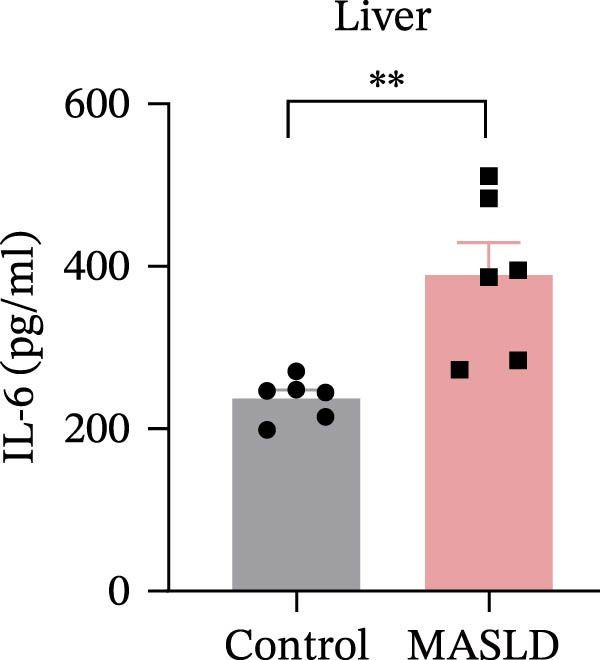
(H)

**Figure:**
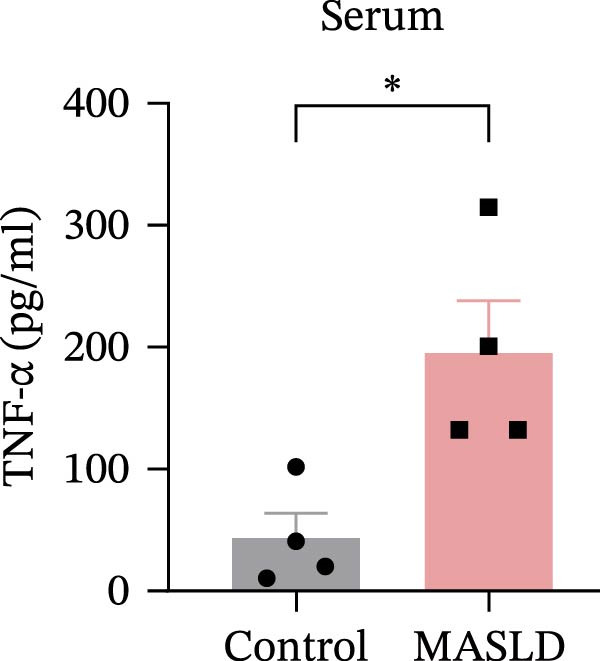
(I)

**Figure:**
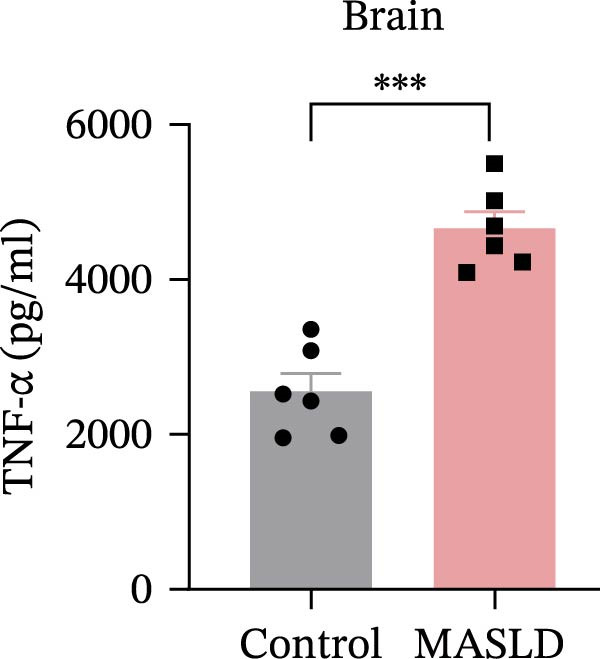
(J)

**Figure:**
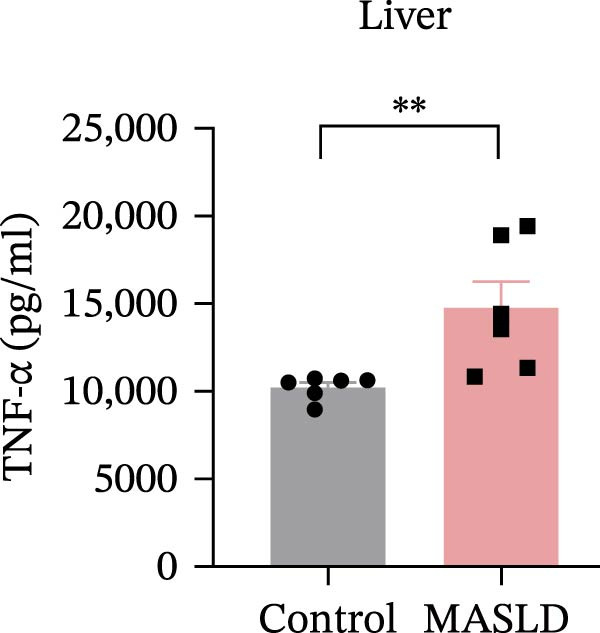
(K)

**Figure:**
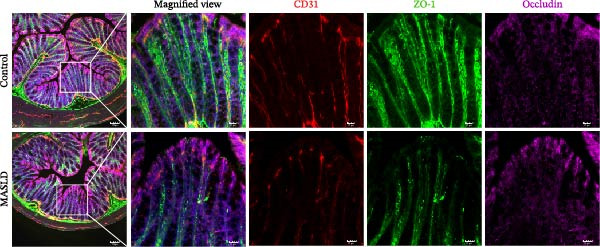
(L)

**Figure:**
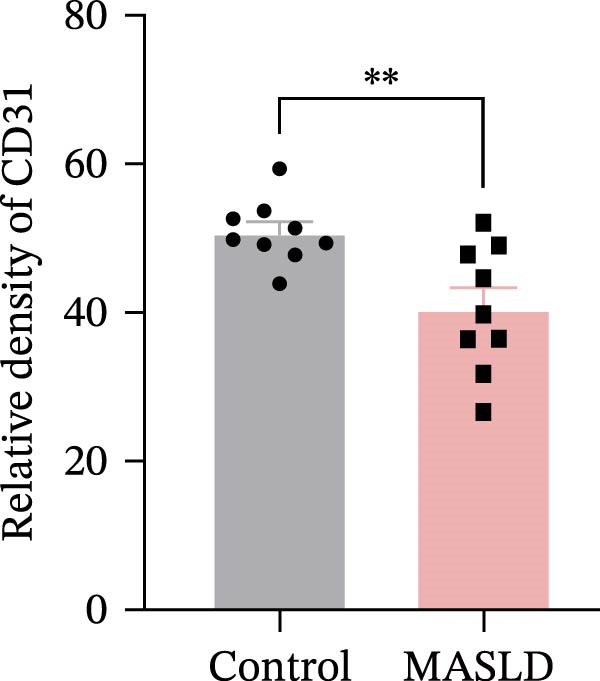
(M)

**Figure:**
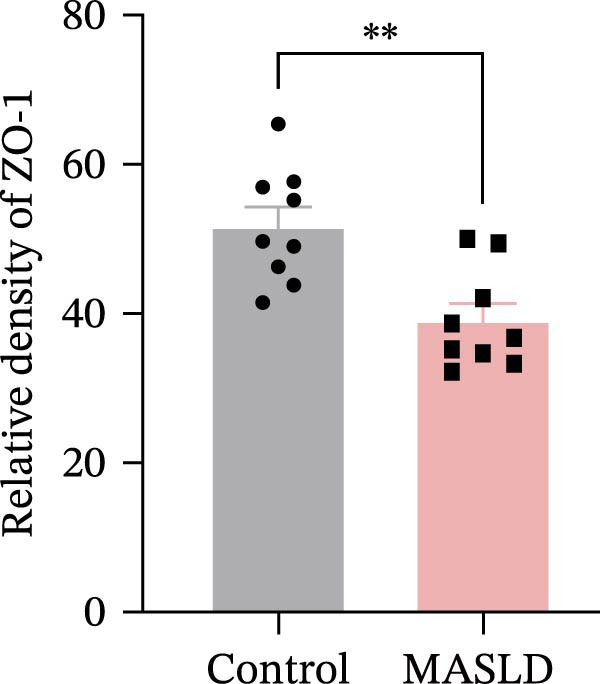
(N)

**Figure:**
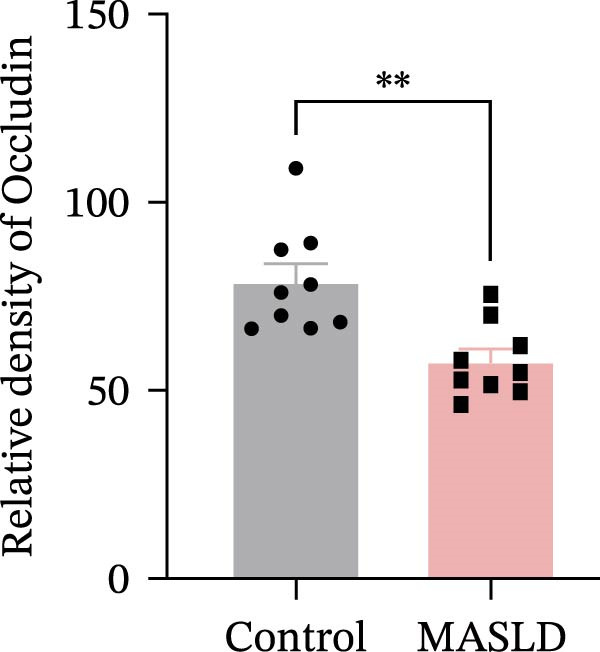
(O)

To further characterize liver pathology, we performed histological analysis using H&E staining. Livers from HFD–fed mice exhibited diffuse macrovesicular steatosis, with large lipid droplets occupying hepatocyte cytoplasm, accompanied by hepatocyte ballooning, cell rounding, and significant enlargement (Figure 4C). These qualitative histological findings were fully consistent with quantitative imaging assessments (Figure 4E), validating the presence of advanced hepatic steatosis and hepatocellular injury.

Beyond hepatic lipid deposition, HFD feeding triggered a robust systemic inflammatory response, a key driver of MASLD–mediated extrahepatic effects. ELISA measurements revealed significant elevations in the cytokines TNF‐α and IL‐6 across serum, liver, and brain tissues (Figure 4F–K), highlighting widespread inflammatory activation. These cytokines are well‐recognized mediators capable of crossing—or disrupting—biological barriers and amplifying inflammation in distant organs, including the CNS.

Since systemic inflammation is often accompanied by intestinal barrier dysfunction, we evaluated the integrity of intestinal epithelial and vascular junctions. Immunofluorescence staining of colonic tissue revealed that the tight junction proteins ZO‐1 and Occludin displayed continuous, well‐organized linear localization in control mice, whereas HFD‐fed mice exhibited markedly reduced fluorescence intensity, disrupted continuity, and fragmented distribution patterns (Figure 4L–O). In parallel, the endothelial junction marker CD31 showed significantly diminished staining in the intestinal vasculature of MASLD mice, indicating compromised vascular integrity.

Collectively, these alterations demonstrate that HFD–induced MASLD disrupts both epithelial and endothelial compartments of the intestinal barrier. Such impairment facilitates the translocation of gut‐derived bacteria (e.g., gram‐negative species) and microbial metabolites including lipopolysaccharide (LPS) into the systemic circulation—mechanisms known to aggravate systemic inflammation and contribute to neuroinflammatory processes through the gut–liver–brain axis [17].

### 3.4. MASLD Model Mice Exhibit Anxiety‐Like Behavior and Memory Impairment

To determine whether MASLD–associated metabolic and inflammatory disturbances extend to functional alterations in the CNS, we conducted a series of behavioral assays assessing anxiety‐like states and memory capability. General locomotor activity remained intact in MASLD mice, as indicated by comparable total distance traveled and average exploration speed in the OF test (Figure 5A–C). Thus, observed behavioral differences were not attributable to impaired mobility.

Figure 5Anxiety‐like behaviors and cognitive impairments in MASLD Mice. (A) The OF test representative trajectory maps. (B) Distance, (C) speed, and (D–F) time spent by the control group and MASLD mice exploring the center zone, peripheral zone, and corner zone of the arena, assessed by the OF test. (G) Y‐Maze representative trajectory map. (H) Speed, (I) distance, (J, K) proportion of the number of explorations by the control group and MASLD mice exploring the novel arm and familiar arm of the arena, and (L, M) time spent by the control group and MASLD mice exploring the novel arm and familiar arm of the arena, assessed by the Y‐maze test (*n* = 6–9 per group). Data are presented as means ± SD.  ^∗^ *p* < 0.05,  ^∗∗^ *p* < 0.01 vs. control. Two‐tailed and unpaired Student’s *t*‐test for all data.

**Figure:**
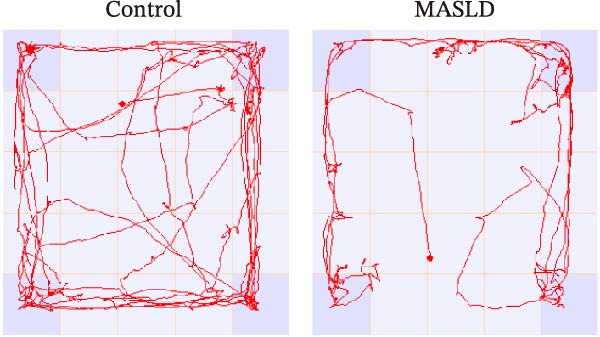
(A)

**Figure:**
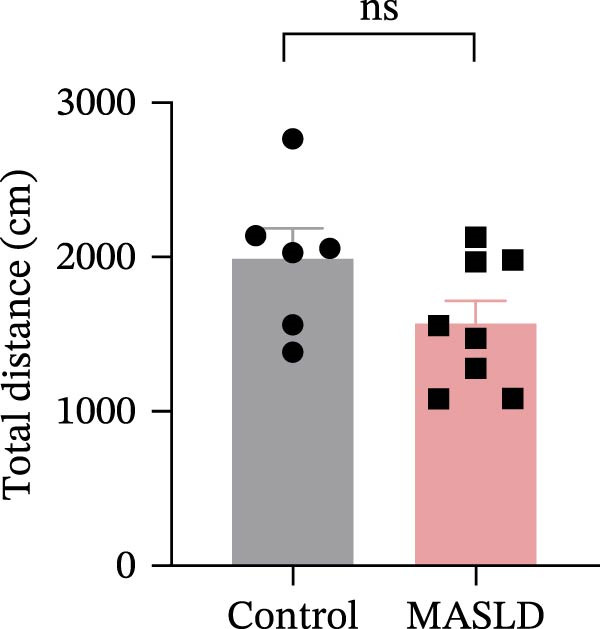
(B)

**Figure:**
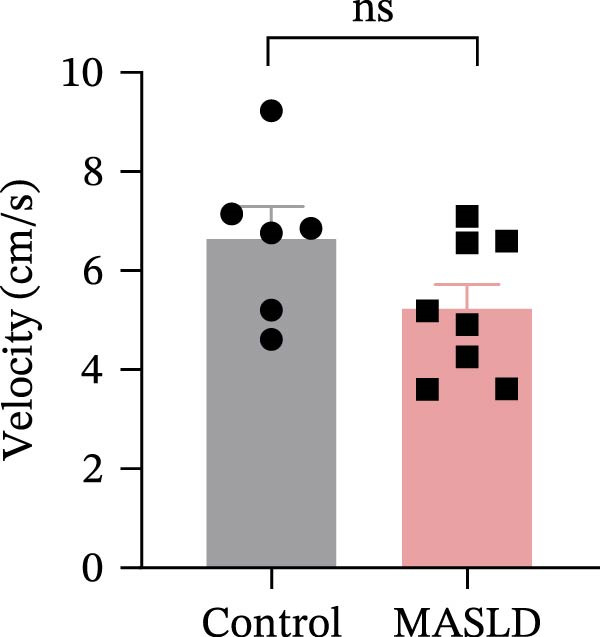
(C)

**Figure:**
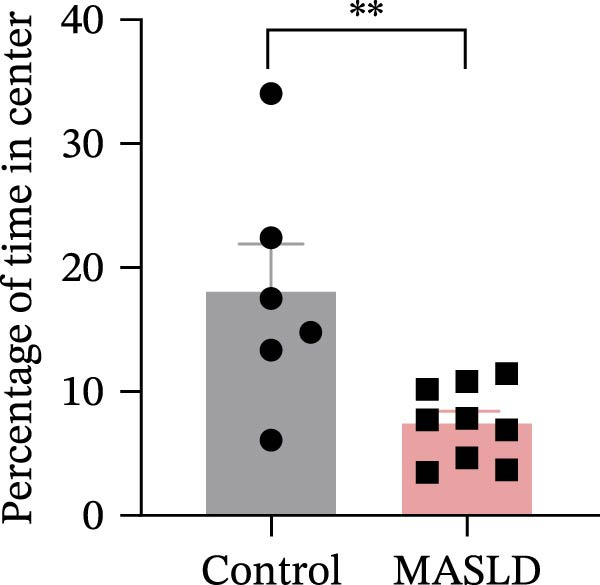
(D)

**Figure:**
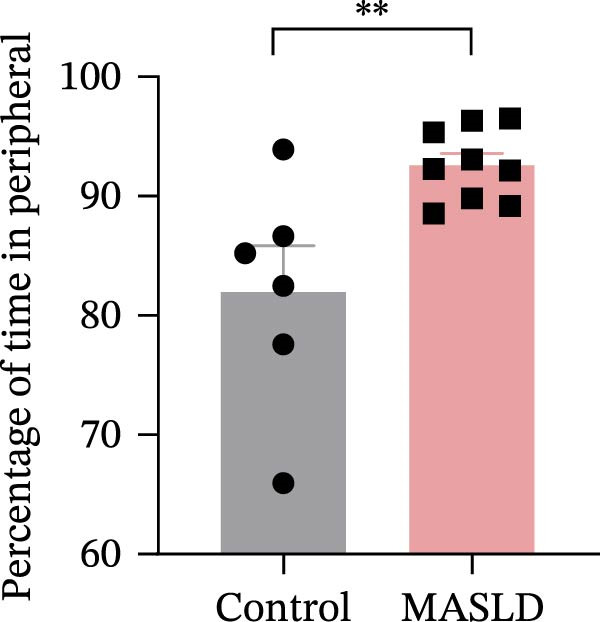
(E)

**Figure:**
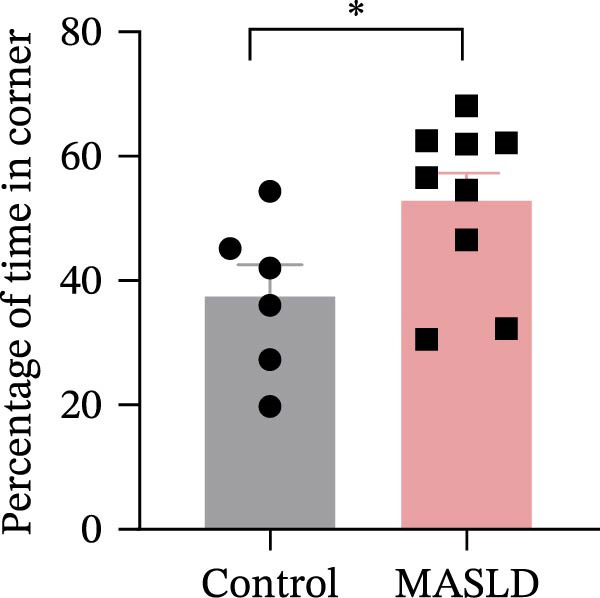
(F)

**Figure:**
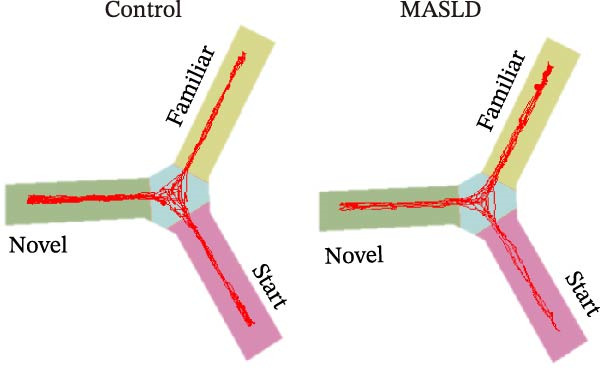
(G)

**Figure:**
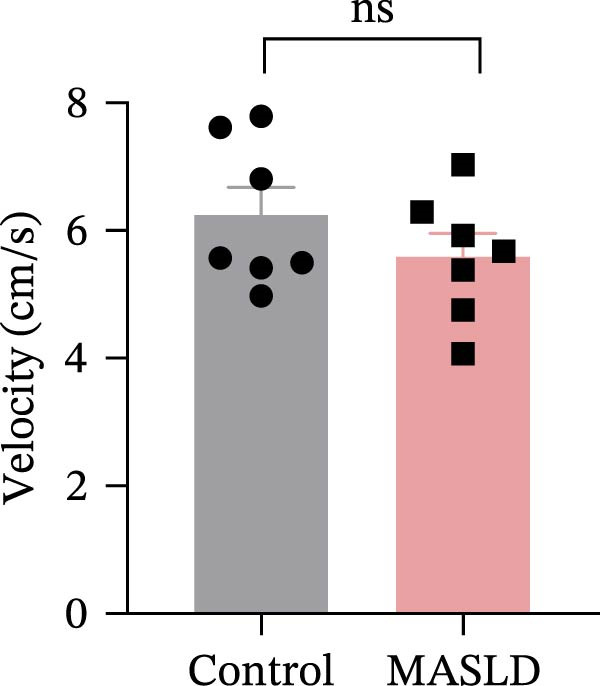
(H)

**Figure:**
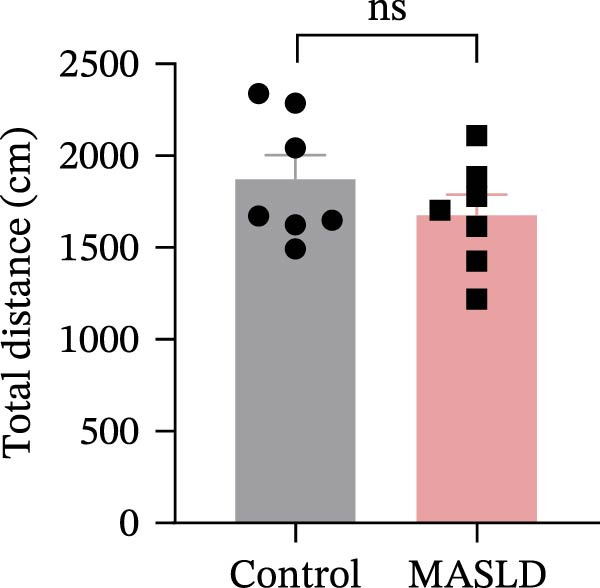
(I)

**Figure:**
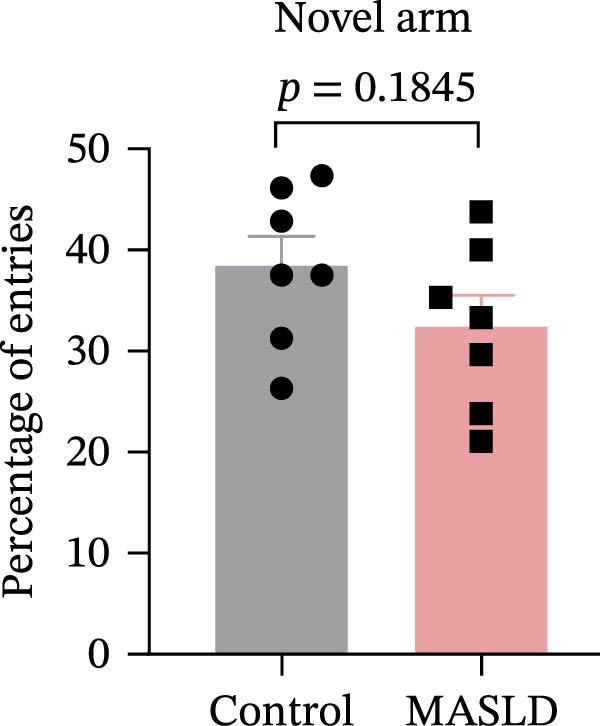
(J)

**Figure:**
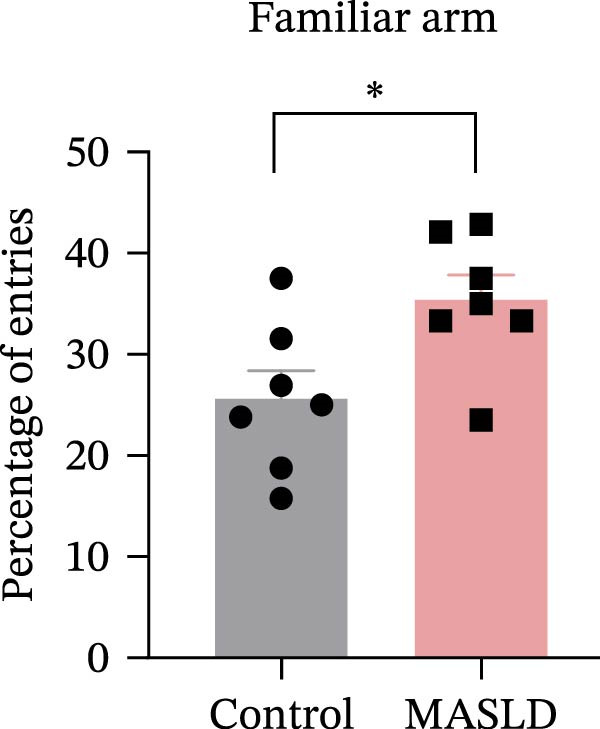
(K)

**Figure:**
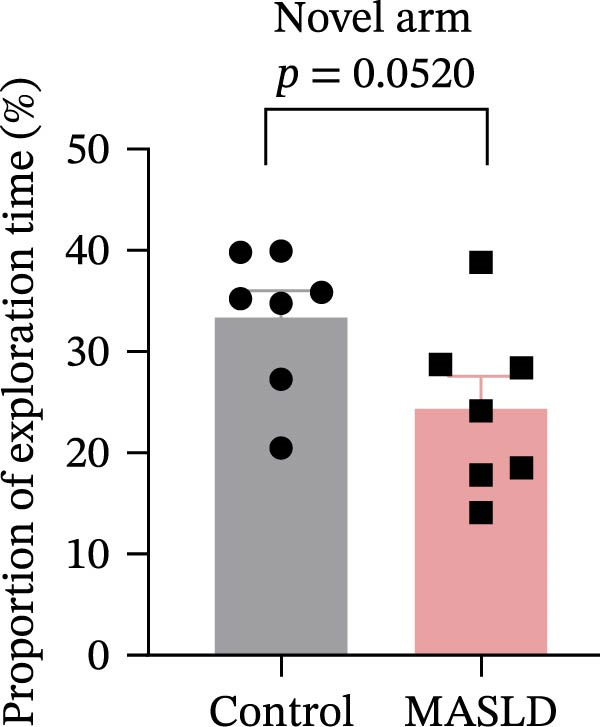
(L)

**Figure:**
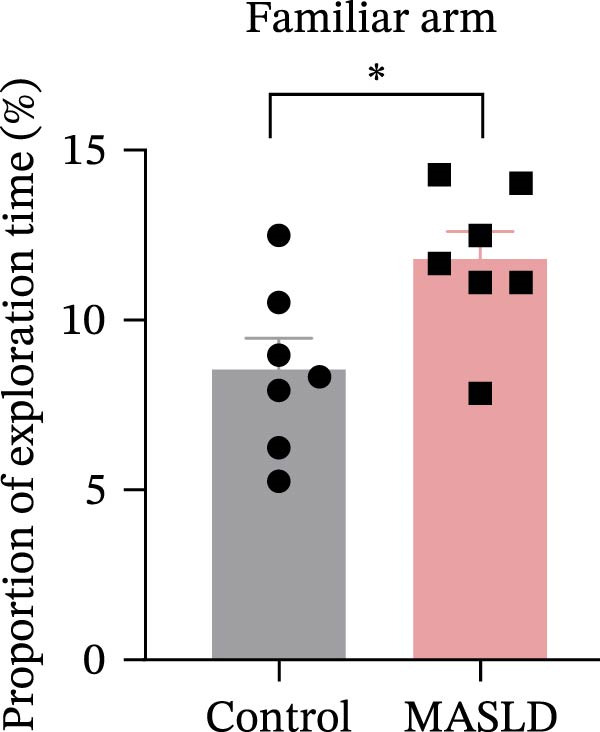
(M)

Despite preserved locomotion, MASLD mice displayed marked anxiety‐like behaviors. Compared with control animals, they spent 58% less time in the central zone (*p* < 0.01), and 13% more time in peripheral areas (*p* < 0.01), and demonstrated a 1.65‐fold increase in corner occupancy (*p* < 0.05; Figure 5D–F). This pattern reflects heightened thigmotaxis—a well‐established marker of anxiety in rodents [18], suggesting that MASLD induces emotional dysregulation likely driven by systemic and neuroinflammatory changes.

Cognitive function was evaluated using the Y‐maze spontaneous alternation paradigm, which probes working memory. Locomotor parameters remained unchanged between groups (Figure 5G–I), confirming cognitive measures were not confounded by reduced activity. MASLD mice made 15% fewer entries into the novel arms (*p* = 0.1845) and a 1.38‐fold more revisits to familiar arms (*p* < 0.05; Figure 5J,K). Temporal exploration patterns similarly indicated impaired novelty recognition: MASLD mice spent 40% more time in familiar arms (*p* < 0.05) and 27% less time exploring the novel arm (*p* = 0.052) compared to normal diet controls (Figure 5L,M). Although some measures approached but did not reach statistical significance, the overall behavioral profile consistently points toward deficits in working memory.

Together, these findings demonstrate that MASLD induces selective neurobehavioral abnormalities characterized by increased anxiety‐like behavior and compromised cognitive function, independent of general motor impairment. These behavioral outcomes align with the observed MR analysis, and provide functional evidence for MASLD‐related disruption of neural circuits.

### 3.5. MASLD–Associated Cytokines Induce Mitochondrial Dysfunction and Promote Neuronal Apoptosis

Given the pronounced anxiety‐like behavior and memory impairment observed in MASLD mice, we next examined whether hippocampal neuronal integrity was compromised. Neurohistological analysis revealed substantial structural alterations within the DG. MASLD mice exhibited a marked 30% reduction in NeuN‐positive neuronal density (*p* < 0.01), accompanied by a 26 % expansion of DG width (*p* < 0.001), indicating neuronal loss and regional tissue remodeling (Figure 6A–C). Remaining neurons displayed abnormal spatial organization, including increased neuronal spacing suggestive of disrupted hippocampal circuitry. Immunofluorescence staining of MAP2 further demonstrated pronounced neuronal pathology. MASLD mice exhibited a significant reduction in MAP2‐positive dendritic area (*p* < 0.05), and dendrites showed fragmentation, diminished branching, and disorganized morphology (Figure 6D, E), consistent with cytoskeletal destabilization and impaired synaptic architecture.

Figure 6MASLD induces hippocampal neuronal degeneration in vivo and cytokine‐mediated mitochondrial dysfunction and apoptosis in HT22 cells in vitro. (A) Representative confocal maximum Z‐projection images showing DAPI and NeuN^+^ neurons in the DG region. (B) Quantification of DG width and (C) NeuN^+^ neuronal density. (D) Representative confocal images of MAP2‐labeled dendritic structures in hippocampal neurons. (E) Quantification of relative MAP2 fluorescence intensity. (F) Representative TUNEL staining of HT22 cells treated with vehicle (control), IL‐6, or TNF‐α. From top to bottom are the control group, the IL‐6 administration intervention group, and the TNF‐α administration intervention group. (G) Quantification of TUNEL‐positive apoptotic area. (H) Representative images of cleaved‐caspase‐3 staining in control, IL‐6‐treated, and TNF‐α‐treated HT22 cells. Arrows indicate regions with cleaved‐caspases signals. (I) Representative images of mitochondrial staining (MitoTracker) under the same treatment conditions. The data are presented as mean ± standard deviation. Compared with the control operation group (control),  ^∗^ *p* < 0.05,  ^∗∗^ *p* < 0.01,  ^∗∗∗^ *p* < 0.001. One‐way analysis of variance (one‐way ANOVA) was employed for the statistical analysis of all data.

**Figure:**
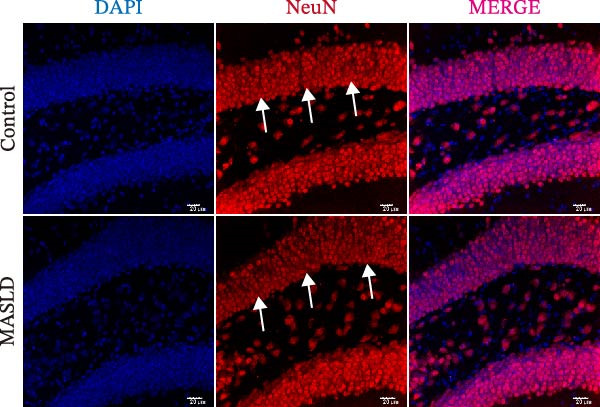
(A)

**Figure:**
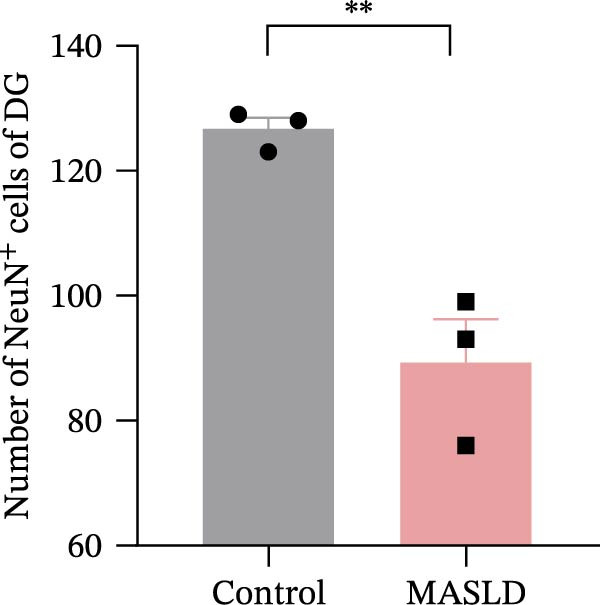
(B)

**Figure:**
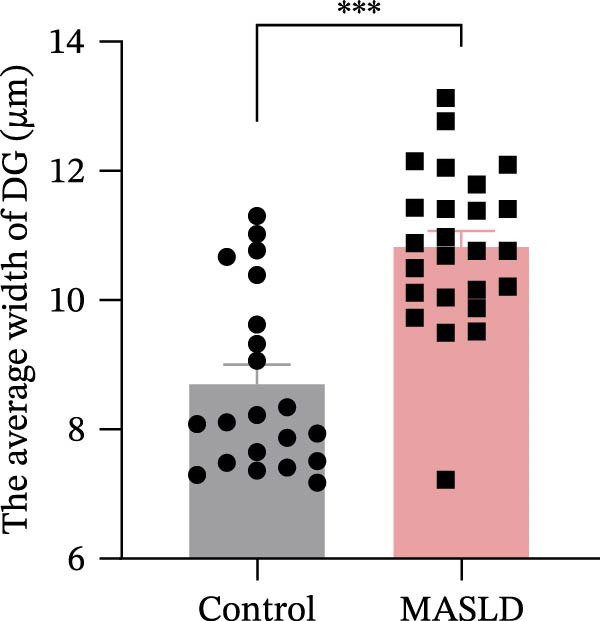
(C)

**Figure:**
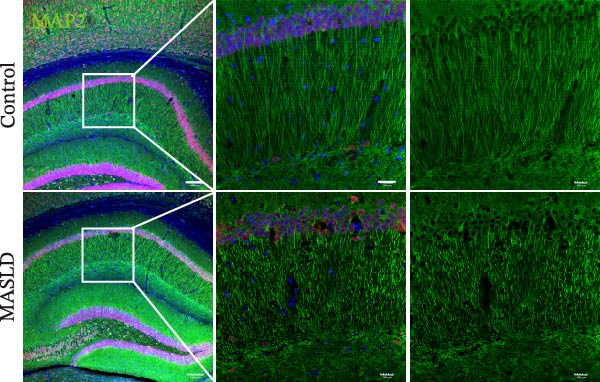
(D)

**Figure:**
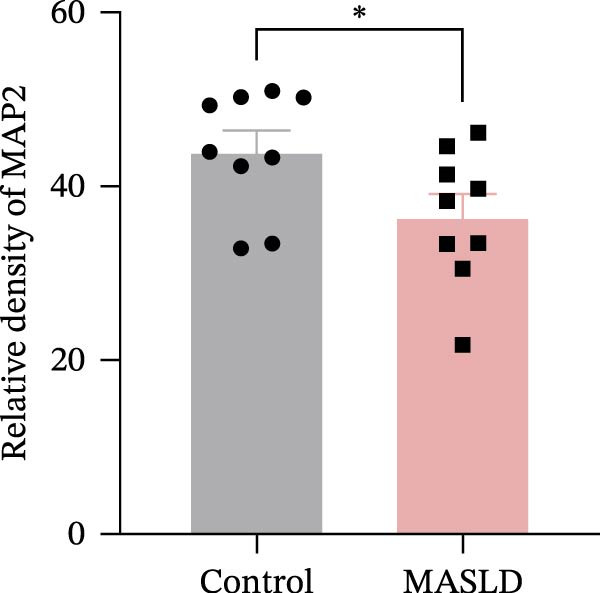
(E)

**Figure:**
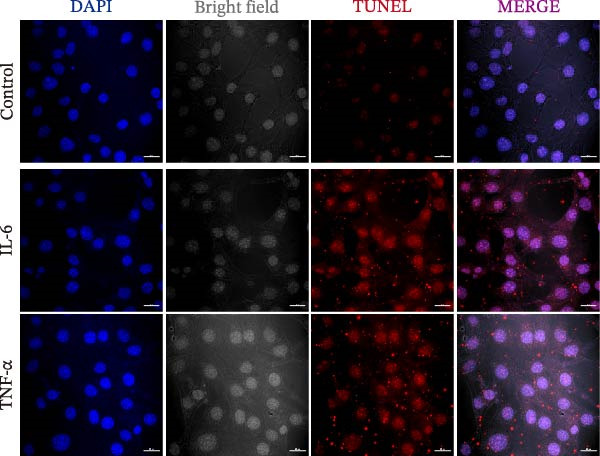
(F)

**Figure:**
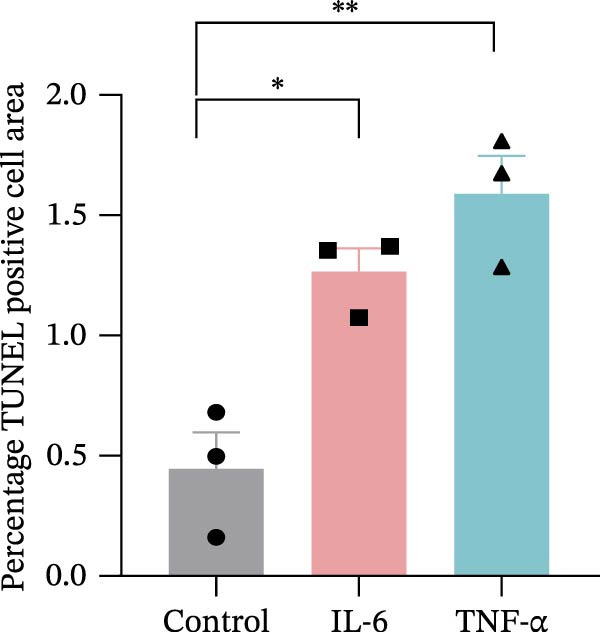
(G)

**Figure:**
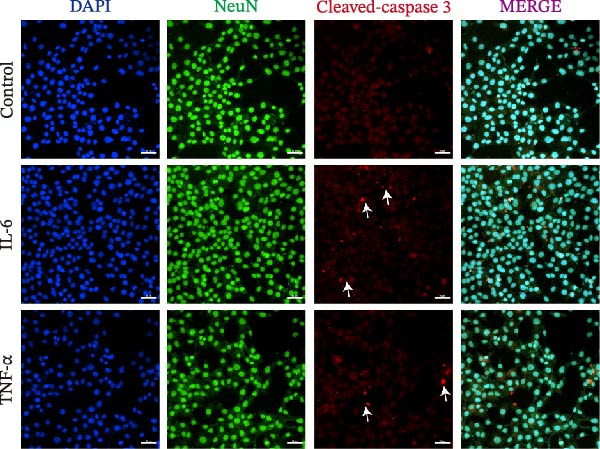
(H)

**Figure:**
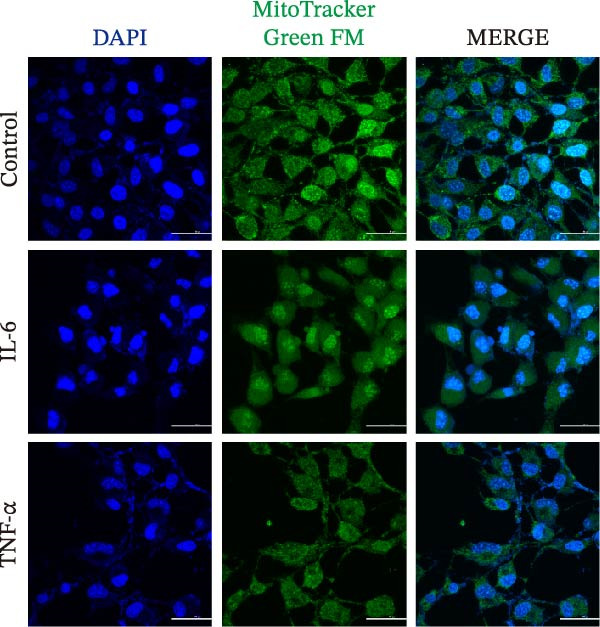
(I)

To investigate potential mechanisms linking systemic inflammation in MASLD to neuronal injury, we examined the direct effects of MASLD–elevated cytokines (IL‐6 and TNF‐α) on TH22 neuronal cells. Both cytokines markedly increased neuronal apoptosis, with TUNEL‐positive cell percentages rising 1.84‐fold following IL‐6 treatment and 2.56‐fold following TNF‐α treatment (Figure 6F, G). These findings were supported by increased cleaved‐caspase‐3 immunoreactivity (Figure 6H), confirming activation of apoptotic pathways. In addition, mitochondrial analysis revealed that cytokine exposure reduced Mito Tracker Green fluorescence by 50%, indicative of mitochondrial dysfunction (Figure 6I).

Together, these findings provide a mechanistic framework in which MASLD‐associated systemic inflammation promotes neuronal apoptosis through mitochondrial impairment, contributing to the hippocampal neurodegeneration underlying behavioral deficits.

### 3.6. MASLD Triggers Astrocytic Remodeling and Shifts Microglia Toward a Pro‐Inflammatory Phenotype in the Hippocampus

Given that neuronal degeneration often occurs against the backdrop of glial cell dysregulation, we next evaluated the morphology of astrocytes and microglia in the hippocampus. GFAP immunostaining revealed marked astrocyte remodeling in MASLD mice. Although the total GFAP coverage area was unchanged, astrocytes displayed reduced soma volume, accompanied by increased process number and elongation (Figure 7A–D)—a structural phenotype characteristic of reactive astrogliosis. This form of glial activation is commonly associated with neuroinflammatory stress and altered synaptic homeostasis [19].

Figure 7MASLD mice display pronounced astrocytic and microglial morphological alterations in the hippocampus. (A) Representative confocal maximum Z‐projection and 2D images of GFAP^+^ astrocytes in the hippocampal. (B) Quantification of astrocyte soma area, (C) number of GFAP^+^ branches per cell, and (D) length of the longest astrocytic branch (*n* = 3–4 per group). (E) Representative confocal maximum Z‐projection and 3D‐rendered images of Iba^+^ microglia in the hippocampal. (F) Quantification of microglial soma area, (G) total filament length, and (H) percentage area occupied by Iba^+^ signal (*n* = 3–4 per group). The data are presented as mean ± standard deviation. Compared with the Control operation group (control),  ^∗^ *p* < 0.05,  ^∗∗^ *p* < 0.01,  ^∗∗∗^ *p* < 0.001. A two‐tailed and unpaired Student’s *t*‐test was used for all data.

**Figure:**
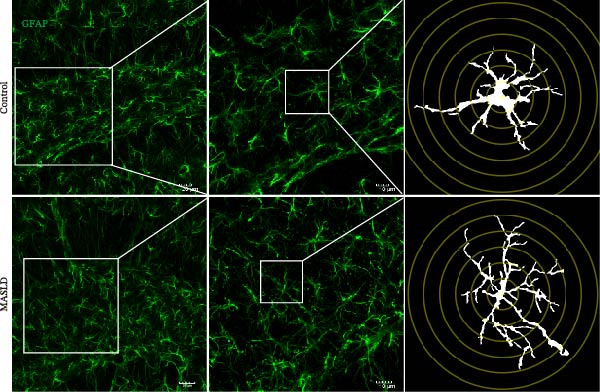
(A)

**Figure:**
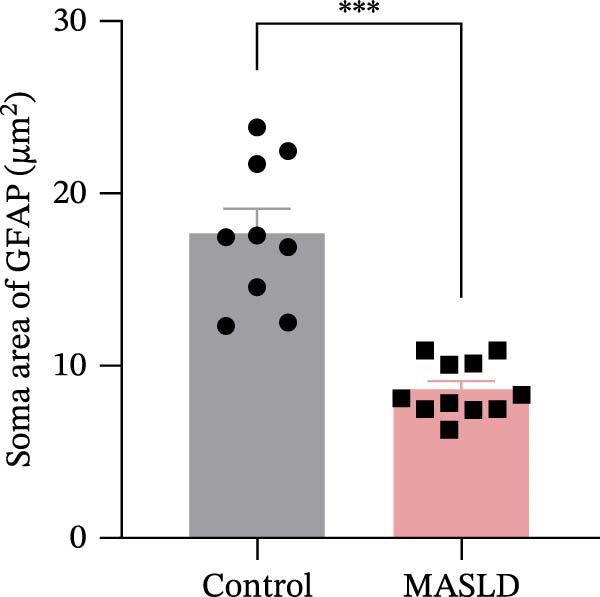
(B)

**Figure:**
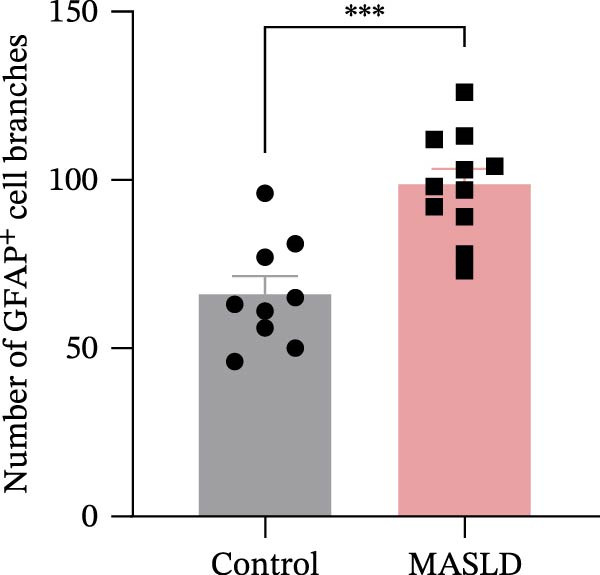
(C)

**Figure:**
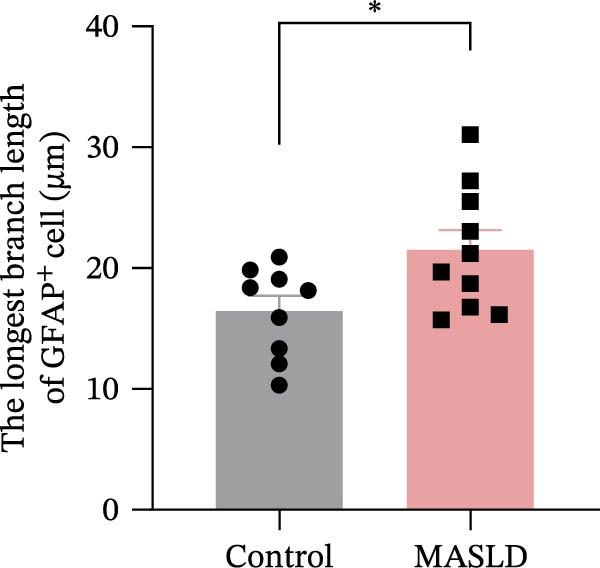
(D)

**Figure:**
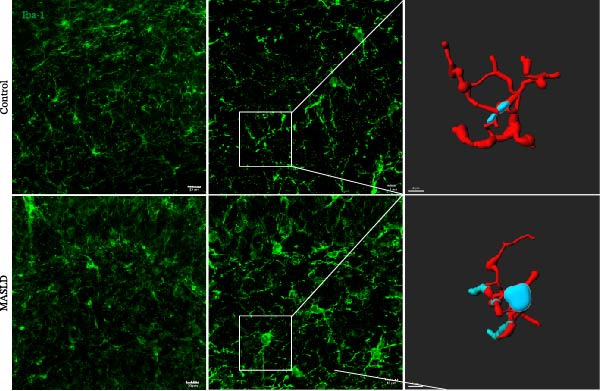
(E)

**Figure:**
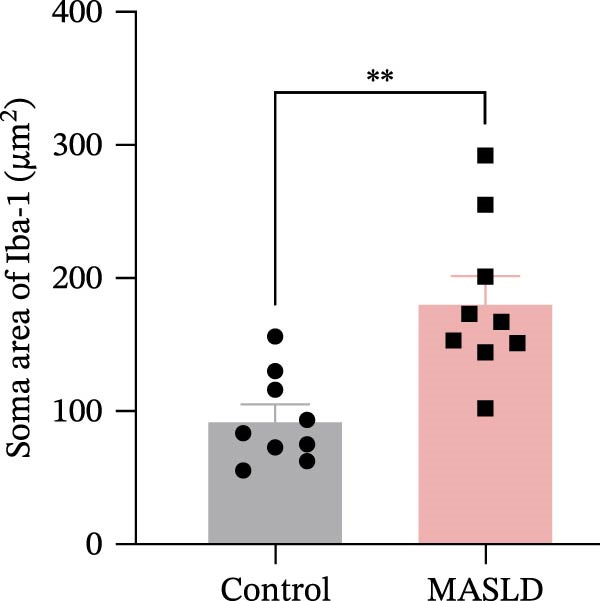
(F)

**Figure:**
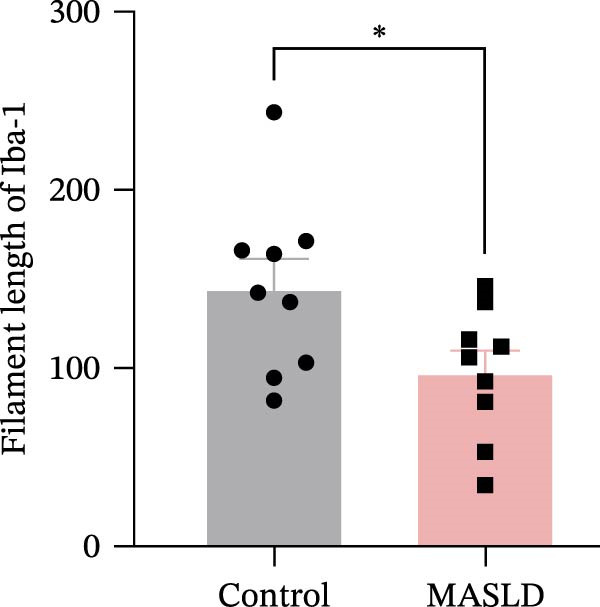
(G)

**Figure:**
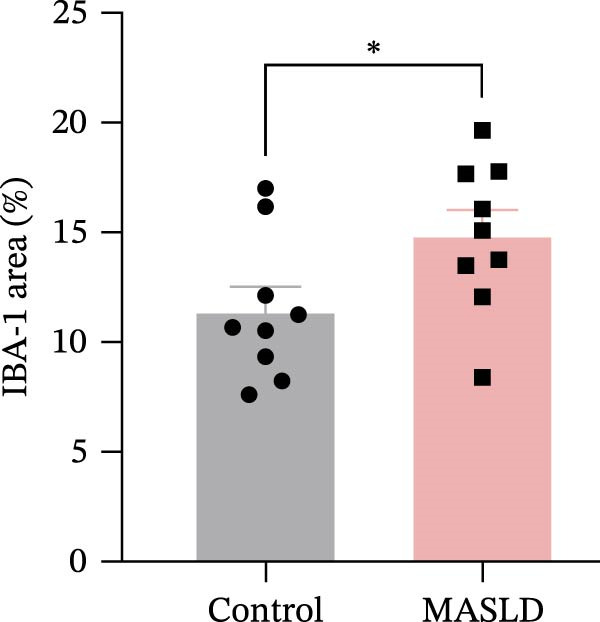
(H)

Microglia exhibited complementary pathological changes. Iba‐1 staining showed that MASLD induced a shift toward an M1‐like pro‐inflammatory phenotype, marked by 50% enlargement of the soma, reduced branching complexity, and shortened processes (Figure 7E–H). Additionally, microglial territorial coverage expanded by 25%, consistent with migration and clustering at inflammatory sites.

These glial abnormalities were most pronounced in the molecular layers of CA1 and the DG regions critical for synaptic integration and memory encoding. The combination of reactive astrogliosis and microglial pro‐inflammatory polarization provides strong evidence that MASLD induces a chronic neuroinflammatory environment that likely contributes to the cognitive and emotional dysfunction observed in behavioral assays.

### 3.7. Transcriptomic Analysis of Key Genes in the Hippocampal Inflammatory Response Affected by MASLD in Mice

To identify molecular pathways underlying MASLD–induced neuroinflammation and cognitive impairment, we performed RNA sequencing on hippocampal tissue. Pearson correlation analysis confirmed high intra‐ and intergroup reproducibility (*r* >0.94), ensuring robust transcriptomic comparisons (Figure 8A).

Figure 8MASLD induces significant alterations in hippocampal gene expression and functional pathway activity. (A) Pearson correlation heatmap illustrating sample‐to‐sample transcriptomic similarity. (B) Volcano plot showing differentially expressed genes (DEGs) between MASLD and control groups; red indicates upregulated genes and blue indicates downregulated genes. (C) Heatmap of the top 10 DEGs, with *n* = 3 control samples (pink) and *n* = 4 MASLD samples (sky blue). (D) GO enrichment analysis, categorized into biological process (BP, green), cellular component (CC, yellow), and molecular function (MF, purple). (E) KEGG enrichment analysis showing MASLD‐associated functional pathway changes; dot size corresponds to the number of DEGs, and dot color (red to blue) represents the *p*‐Values (red represents smaller *p*‐Values, while blue represents larger *p*‐Values). (F) Integrative correlation analysis of GO and KEGG pathways: the left panel shows GO‐enriched pathways, the middle panel shows co‐enriched genes, and the right panel shows KEGG‐enriched pathways. (G–J) Relative mRNA expression levels of *LCN2* (G), *TCF7L2* (H), *AQP1* (I), and *Cldn1* (J) in the MASLD and control hippocampal tissues (*n* = 3–4 per group). The data are presented as mean ± standard deviation. Compared with the control group,  ^∗^ *p* < 0.05,  ^∗∗^ *p* < 0.01,  ^∗∗∗^ *p* < 0.001. A two‐tailed, unpaired Student’s *t*‐test was used for all statistical analyses.

**Figure:**
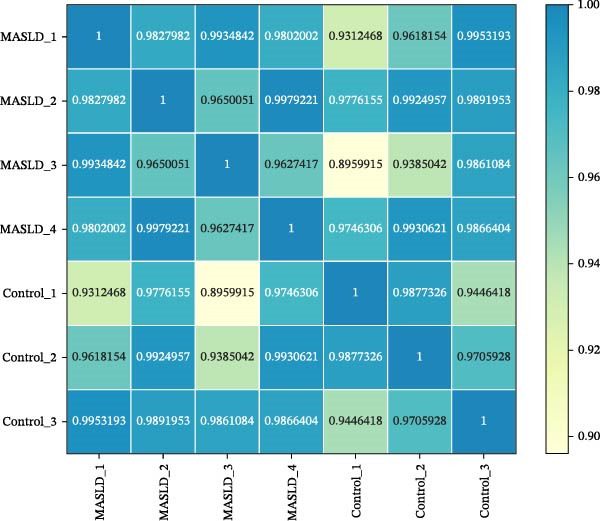
(A)

**Figure:**
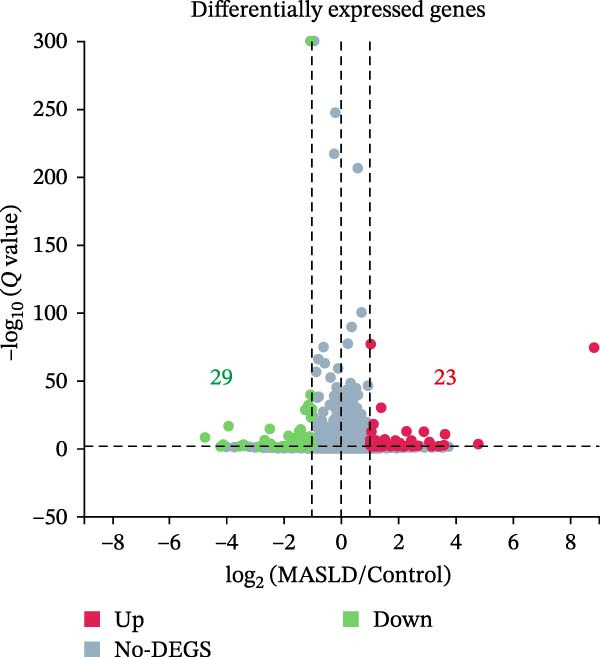
(B)

**Figure:**
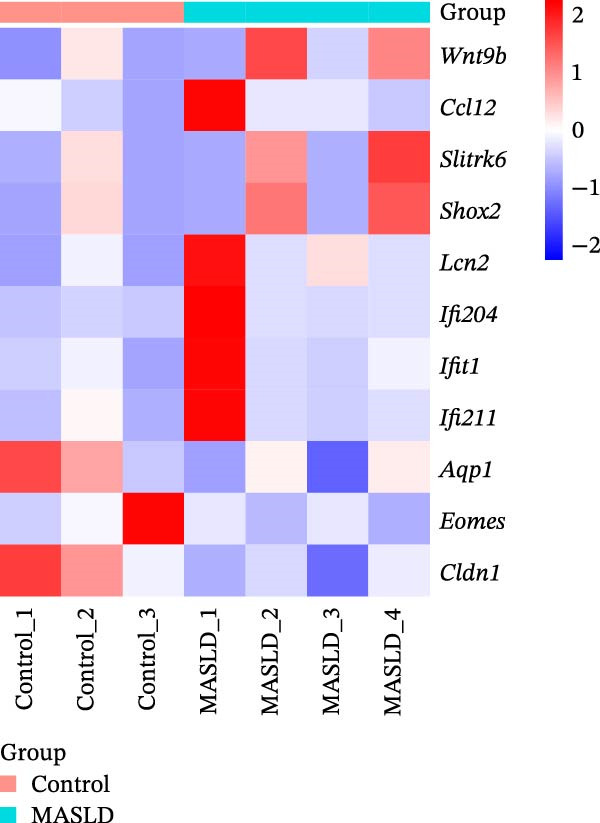
(C)

**Figure:**
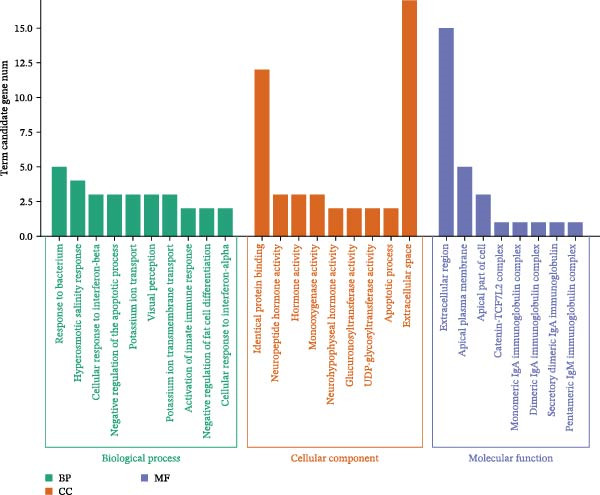
(D)

**Figure:**
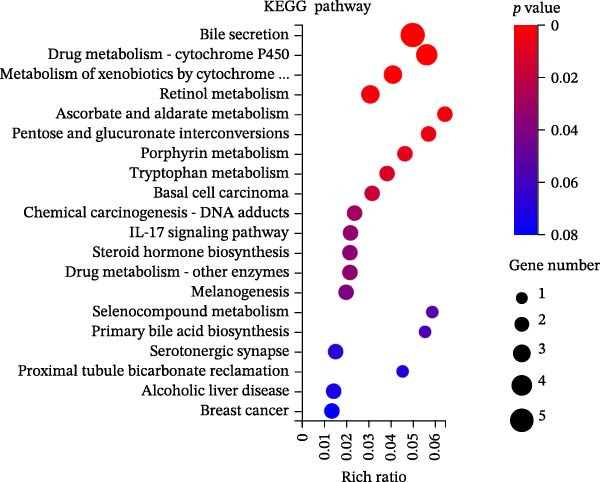
(E)

**Figure:**
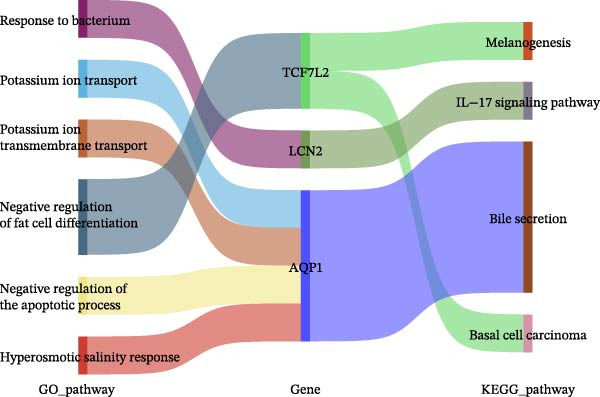
(F)

**Figure:**
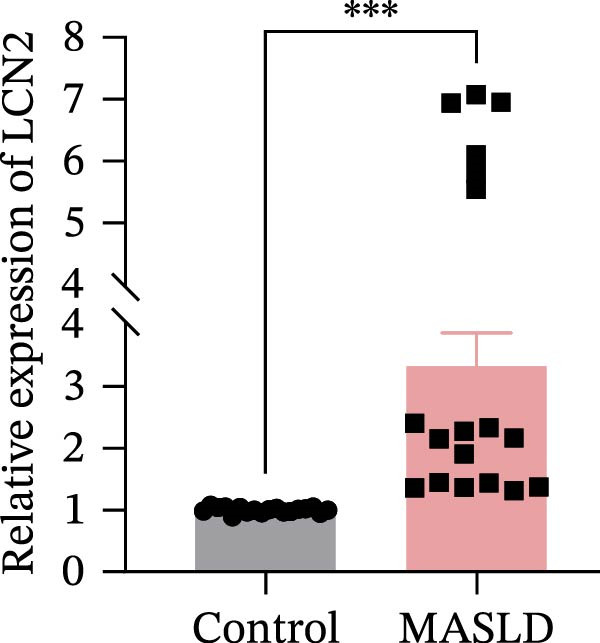
(G)

**Figure:**
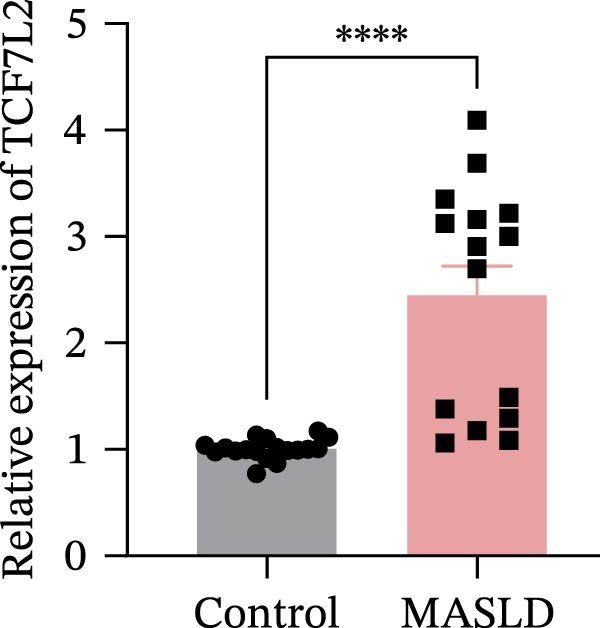
(H)

**Figure:**
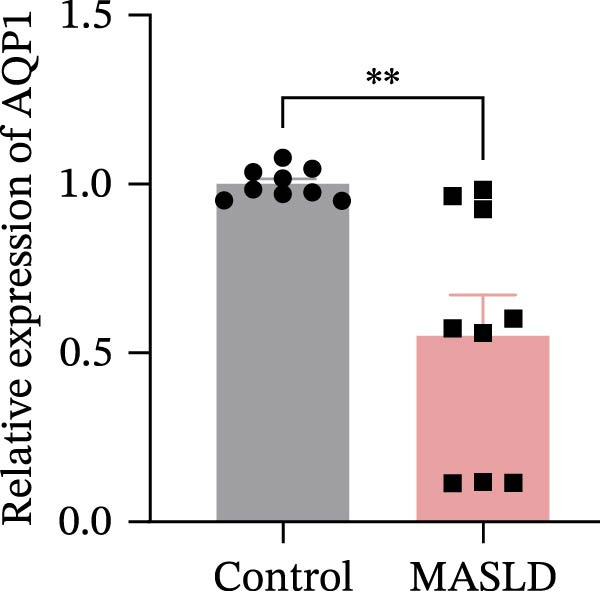
(I)

**Figure:**
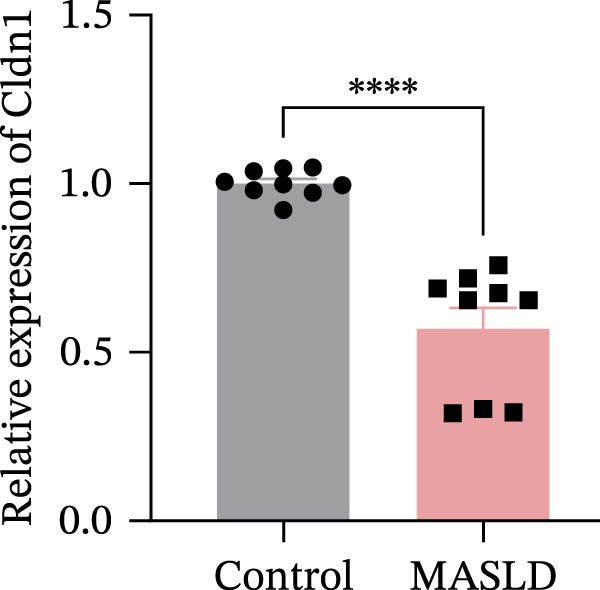
(J)

Differential expression analysis identified 52 significantly alter genes (|log_2_FC| >1, *p* < 0.05), including 23 upregulated and 29 downregulated transcripts in MASLD mice (Figure 8B). Upregulated genes included several inflammatory mediators (e.g., *Wnt9b*, *Ccl12*, *Slitrk6*, *Shox2*, *Lcn2*, *Ifi204*, *Ifit1*, and *Ifi211*), while genes involved in metabolic regulation and blood‐brain barrier integrity (e.g., *Aqp1*, *Eomes*, and *Cldn1*) were downregulated (Figure 8C).

GO enrichment analysis revealed that differentially expressed genes were primarily associated with immune and inflammatory responses, cell apoptosis, metabolic regulation, extracellular matrix organization, and transcriptional control (Figure 8D). KEGG pathway analysis further indicated enrichment of lipid metabolism, bile secretion, and immune signaling pathways (Figure 8E), highlighting the convergence of metabolic and inflammatory dysregulation in MASLD–driven hippocampal injury.

Integrated GO–KEGG correlation analysis identified *TCF7L2*, *LCN2*, and *AQP1* as key nodes linking multiple dysregulated pathways (Figure 8F). qPCR validation confirmed significant changes in the expression of *TCF7L2*, *LCN2*, *AQP1*, and *Cldn1* (Figure 8G–J), supporting transcriptomic findings.

Collectively, these results reveal that MASLD induces coordinated transcriptional alterations affecting immune activation, lipid metabolism, and cellular stress pathways in the hippocampus—molecular changes that likely contribute to the observed glial activation, neuronal injury, and behavioral deficits.

## 4. Discussion

In this study, we combined genetic epidemiology, experimental neuroscience, and hippocampal transcriptomics to establish a mechanistic link between metabolic dysfunction in MASLD and brain injury leading to cognitive and emotional deficits. MR analysis provided the first line of evidence, demonstrating that genetically predicted MASLD is causally associated with impaired cognitive performance—including deficits in processing accuracy, reading and spelling abilities, and phonological awareness—as well as increased anxiety‐related phenotypes. These findings suggested that MASLD may exert direct effects on the CNS, prompting further biological investigation.

Using the HFD mouse model, we confirmed that MASLD induces profound systemic inflammation, marked by increased circulating IL‐6 and TNF‐α, paralleling clinical observations [20]. Importantly, these inflammatory signals were accompanied by specific neurobiological abnormalities: hippocampal neuronal loss, dendritic degeneration, reactive astrocytosis, microglial polarization toward a pro‐inflammatory phenotype, and behavioral deficits in anxiety‐like behavior and spatial memory. Together, these results identify MASLD as a systemic metabolic disorder capable of driving hippocampal dysfunction through peripheral‐to‐central inflammatory signaling.

### 4.1. Inflammatory Cytokines as Key Mediators of MASLD–Induced Neurodegeneration

Mechanistic in vitro experiments demonstrated that IL‐6 and TNF‐α—two cytokines markedly elevated in MASLD—directly impair neuronal health by inducing mitochondrial dysfunction and activating the apoptotic cascade. These findings align with previous studies showing that chronic inflammation and oxidative stress are central drivers of neuronal vulnerability and neurodegeneration [21, 22]. In vivo, the hippocampus of MASLD mice exhibited hallmark features of neuroinflammatory damage, including dendritic fragmentation, neuronal apoptosis, and glial cell remodeling. Notably, the astrocytic and microglial morphological changes observed here resemble those seen in Alzheimer’s and Parkinson’s diseases, suggesting that MASLD may engage shared inflammatory pathways implicated in classical neurodegenerative conditions [20, 23].

Astrocytes and microglia are critical regulators of synaptic function, glutamate buffering, neurotrophic support, and extracellular ion homeostasis [24, 25]. Disruption of these functions disproportionately affects hippocampal subregions such as CA1, CA3, and DG—areas central to learning and memory [26–28]. The convergence of neuronal injury and glial dysfunction, therefore, provides a robust cellular basis for the observed behavioral abnormalities [29, 30].

### 4.2. Potential Contribution of the Gut–Liver–Brain Axis

In addition to inflammatory cytokines, our findings point toward impaired intestinal barrier integrity as another contributor to MASLD–associated neurocognitive impairment. Growing evidence supports a bidirectional gut–liver–brain axis in which intestinal dysbiosis and barrier dysfunction enhance systemic exposure to bacterial metabolites (e.g., LPS), amplifying hepatic inflammation and promoting neuroimmune activation [31, 32]. Through vagus nerve signaling, microbial metabolites, and immune mediators, alterations in gut ecology can influence CNS inflammation, synaptic function, and neurogenesis [19]. Furthermore, dysbiosis promotes the abnormal proliferation of intestinal Th17 cells; mucin degradation mediated by these cells further induces central microglial activation, astrogliosis, and aberrant activation of the HPGD/JAK2/STAT3 pathway in the brain, amplifying the inflammatory cascade [33, 34]. Although the current study did not directly interrogate gut microbiota changes, our observations of barrier dysfunction suggest that gut‐derived inflammatory signals may further reinforce the hepatic and neural pathology of MASLD. Future work involving 16S sequencing, fecal microbiota transplantation, and germ‐free models will be essential to clarify these interactions and identify microbiome‐dependent mechanisms contributing to MASLD–related neurocognitive decline.

### 4.3. Transcriptomic Signatures Highlight Converging Metabolic and Inflammatory Pathways

Transcriptomic analysis in this study confirmed that MASLD profoundly alters hippocampal gene expression. Notably, inflammatory genes such as *mTcf7l2*, *Lcn2*, and *Ifi* family members were markedly upregulated, while metabolism‐ and barrier‐related genes, including *AQP1* and *Claudin 1* were significantly downregulated. These transcriptional changes were enriched in pathways regulating immune activation, cell apoptosis, lipid metabolism, extracellular matrix remodeling, and synaptic function.

Several of these genes have well‐established roles in neuroinflammation or neurodegeneration. For example, *TCF7L2* regulates Wnt signaling in astrocytes [35, 36]; *Lcn2* amplifies microglial activation and disrupts blood–brain barrier integrity [37–39]; *Ifi* family genes potentiate interferon signaling and apoptotic cascades [40]. Conversely, reduced *AQP1* may impair cerebrospinal fluid homeostasis and promote neurotoxic protein accumulation [41–43]. Together, these transcriptomic changes mirror the structural and functional impairments observed in the hippocampus, suggesting a multilayered disruption of metabolic, immune, and neurotrophic signaling in MASLD.

### 4.4. Broader Metabolic Mechanisms Linking MASLD and CNS Dysfunction

Although inflammation was the primary focus of mechanistic validation, other pathological features of MASLD likely interact with neuroinflammatory pathways. Insulin resistance—central to MASLD pathogenesis—can impair neuronal glucose uptake, downregulate GLUT3, disrupt mitochondrial metabolism, and compromise synaptic function [44–46]. Lipotoxic intermediates generated in MASLD, such as palmitic acid, can cross the BBB and induce ER stress, oxidative injury, and ferroptosis in CNS cells [47]. Moreover, HFD–induced intestinal barrier disruption facilitates the translocation of LPS and cytokines, promoting hepatic insulin resistance and perpetuating the inflammatory cycle [6]. These interconnected mechanisms form a metabolic–inflammatory network that may further exacerbate CNS vulnerability in MASLD.

Despite these advances, limitations remain. The HFD mouse model recapitulates key metabolic features of MASLD, but it does not fully mimic the fibrosis progression seen in human patients. Furthermore, the HT‐22 immortalized cells have certain limitations, which may make it difficult to extrapolate the research results to in vivo neuroinflammatory response scenarios involving complex neuron–glial interactions. Therefore, future studies should adopt primary rodent neuron cultures, glial cell cultures, and mixed neuron–glial cell culture systems to verify the liver–brain axis mechanism in MASLD; meanwhile, it is necessary to further explore the cross talk mechanisms between astrocytes and neurons (such as BDNF signaling and glutamate cycling) and evaluate the effects of targeted intervention measures for specific inflammatory pathways, such as IL‐6/TNF‐α inhibition and PPAR‐γ activation [48]. Translational efforts should prioritize validating serum inflammatory markers as predictors of cognitive risk in MASLD patients and assessing whether early metabolic interventions mitigate neuropathology in neurodegenerative models.

## 5. Conclusions

MASLD is not merely a hepatic disorder, but a systemic disease with profound neurological consequences. By identifying the key inflammatory, metabolic, and transcriptional mediators of cognitive dysfunction, this study provides integrative evidence that MASLD contributes directly to cognitive impairment through a liver–brain axis mediated by systemic inflammation, neuronal mitochondrial dysfunction, glial activation, and hippocampal transcriptional remodeling. Genetic epidemiology confirmed a causal relationship between MASLD and cognitive/anxiety phenotypes, while in vivo and in vitro experiments delineated the cellular and molecular pathways underlying hippocampal vulnerability. Key genes, including TCF7L2 and LCN2, were identified as central regulators of neuroinflammation and metabolic signaling, highlighting potential therapeutic targets.

**Nomenclature**
MASLD: Metabolic dysfunction–associated steatotic liver disease
MR: Mendelian randomization
HFD: High‐fat diet
DG: Dentate gyrus
CNS: Central nervous system
MASH: Metabolic dysfunction–associated steatohepatitis
OF: Open field
ALT: Alanine aminotransferase
AST: Aspartate aminotransferase
ALP: Alkaline phosphatase
TBIL: Total bilirubin
DBIL: Direct bilirubin
ALB: Albumin
GLB: Globulin
LDL: Low‐density lipoprotein
CHOL: Cholesterol
H&E: Hematoxylin and eosin
IL‐6: Interleukin‐6
TNF‐α: Tumor necrosis factor‐α
WT: Wild‐type
Elisa: Enzyme‐linked immunosorbent assay
*R* ^2^: Orrelation coefficient
CV: Coefficient of variation
RRID: Resource identification initiative ID
IVW: Inverse variance weighting
GO: Gene Ontology
KEGG: Kyoto Encyclopedia of Genes and Genomes
ROs: Ratio of ratios
CIs: Confidence intervals
LPS: Lipopolysaccharide
DEGs: Differentially expression genes
CC: Cellular component
BP: Biological process
MF: Molecular function.

## Author Contributions

All authors contributed to the study conception and design. Conceptualization: Tian‐Tian Peng and Qian Hua. Methodology, formal analysis: Tian‐Tian Peng, Yu Shi, and Rui Yu. Data curation: Jia‐Ni Zhang. Writing – original draft: Tian‐Tian Peng and Yu Shi. Writing – review and editing: Yan Mu, Tong Jin, and Yu‐Xin Nie. Supervision: Yan Tan and Qian Hua. Funding acquisition: Yan Tan, Xu Wang, and Qian Hua.

## Funding

This study was funded by the National Natural Science Foundation of China (Grants 82374175, 82304915, and U21A201401).

## Disclosure

All authors read and approved the final manuscript.

## Ethics Statement

All procedures performed in studies involving animals were in accordance with the ethical standards of the institution at which the studies were conducted and ethical approval was obtained from the Laboratory Animal Center of Beijing University of Chinese Medicine BUCM‐2024070401‐3011.

## Conflicts of Interest

The authors declare no conflicts of interest.

## Supporting Information

Additional supporting information can be found online in the Supporting Information section.

## Supporting information


**Supporting Information 1** Figure S1: The scatter plot of MR results. The more consistent in direction and the smaller the dispersion among the five methods, the greater the consistency in MR results. Figure S2: The single‐SNP plots of MR results. It represents the individual MR analysis results for each SNP. Figure S3: The funnel plots of MR results. Figure S4: The leave‐one‐out forest plots of MR results.

**Supporting Information 2** Table S1: STROBE‐MR checklist of recommended items to address in reports of Mendelian randomization studies. Table S2: The characteristics of the GWAS data sources used in this study. Table S3: SNPs for NAFLD used in MR. Table S4: Other four MR results. Table S5: The results of *Q*‐statistics and horizontal pleiotropy tests.

## Data Availability

The data of this study are available from the corresponding author (Yan Tan, yantan@bucm.edu.cn) upon reasonable request.

## References

[1] Kadi D. , Loomba R. , and Bashir M. R. , Diagnosis and Monitoring of Nonalcoholic Steatohepatitis: Current State and Future Directions, Radiology. (2024) 310, no. 1, 10.1148/radiol.222695. 38226882

[2] Machado M. V. and Cortez-Pinto H. , NAFLD, MAFLD and Obesity: Brothers in Arms?, Nature Reviews Gastroenterology & Hepatology. (2023) 20, no. 2, 67–68, 10.1038/s41575-022-00717-4. 36470966

[3] Giuffrè M. , Merli N. , Pugliatti M. , and Moretti R. , The Metabolic Impact of Nonalcoholic Fatty Liver Disease on Cognitive Dysfunction: A Comprehensive Clinical and Pathophysiological Review, International Journal of Molecular Sciences. (2024) 25, no. 6, 10.3390/ijms25063337, 3337. 38542310 PMC10970252

[4] Hadjihambi A. , Konstantinou C. , and Klohs J. , et al.Partial MCT1 Invalidation Protects Against Diet-Induced Non-Alcoholic Fatty Liver Disease and the Associated Brain Dysfunction, Journal of Hepatology. (2023) 78, no. 1, 180–190, 10.1016/j.jhep.2022.08.008. 35995127

[5] George E. S. , Sood S. , Daly R. M. , and Tan S. Y. , Is There an Association Between Non-Alcoholic Fatty Liver Disease and Cognitive Function? A Systematic Review, BMC Geriatrics. (2022) 22, no. 1, 10.1186/s12877-021-02721-w. PMC8753832 35016619

[6] Meroni M. , Longo M. , Paolini E. , and Dongiovanni P. , A Narrative Review About Cognitive Impairment in Metabolic Dysfunction-Associated Steatotic Liver Disease (MASLD): Another Matter to Face Through a Holistic Approach, Journal of Advanced Research. (2025) 68, 231–240, 10.1016/j.jare.2024.02.007. 38369241 PMC11785580

[7] Peng X. , Zhang X. , and Xu Z. , et al.Peripheral Amyloid-β Clearance Mediates Cognitive Impairment in Non-Alcoholic Fatty Liver Disease, EBioMedicine. (2024) 102, 10.1016/j.ebiom.2024.105079, 105079. 38507874 PMC10965463

[8] Gu L. , Zhu Y. , and Nandi S. P. , et al.FBP1 Controls Liver Cancer Evolution From Senescent MASH Hepatocytes, Nature. (2025) 637, no. 8045, 461–469, 10.1038/s41586-024-08317-9. 39743585 PMC12168545

[9] Cherubini A. , Della Torre S. , Pelusi S. , and Valenti L. , Sexual Dimorphism of Metabolic Dysfunction-Associated Steatotic Liver Disease, Trends in Molecular Medicine. (2024) 30, no. 12, 1126–1136, 10.1016/j.molmed.2024.05.013. 38890029

[10] Meyer J. , Teixeira A. M. , and Richter S. , et al.Sex Differences in Diet-Induced MASLD-are Female Mice Naturally Protected?, Frontiers in Endocrinology. (2025) 16, 10.3389/fendo.2025.1567573, 1567573. 40162312 PMC11949793

[11] Zhang K. , Chang Q. , Li F. , Li Y. , Ding R. , and Yu Y. , The Locus Coeruleus-Dorsal Hippocampal CA1 Pathway is Involved in Depression-Induced Perioperative Neurocognitive Disorders in Adult Mice, CNS Neuroscience & Therapeutics. (2024) 30, no. 2, 10.1111/cns.14406. PMC10848051 37577850

[12] Deacon R. M. and Rawlins J. N. , T-Maze Alternation in the Rodent, Nature Protocols. (2006) 1, no. 1, 7–12, 10.1038/nprot.2006.2, 2-s2.0-33947668266. 17406205

[13] Zheng Y. , Xu C. , and Sun J. , et al.Excitatory Somatostatin Interneurons in the Dentate Gyrus Drive a Widespread Seizure Network in Cortical Dysplasia, Signal Transduction and Targeted Therapy. (2023) 8, no. 1, 10.1038/s41392-023-01404-9. PMC10188524 37193687

[14] Burgess S. and Thompson S. G. , Avoiding Bias From Weak Instruments in Mendelian Randomization Studies, International Journal of Epidemiology. (2011) 40, no. 3, 755–764, 10.1093/ije/dyr036, 2-s2.0-79961177302. 21414999

[15] Xuan Y. , Wu D. , Zhang Q. , Yu Z. , Yu J. , and Zhou D. , Elevated ALT/AST Ratio as a Marker for NAFLD Risk and Severity: Insights From a Cross-Sectional Analysis in the United States, Frontiers in Endocrinology. (2024) 15, 10.3389/fendo.2024.1457598, 1457598. 39253584 PMC11381241

[16] Fondevila M. F. , Novoa E. , and Fernandez U. , et al.Inhibition of Hepatic p63 Ameliorates Steatohepatitis With Fibrosis in Mice, Molecular Metabolism. (2024) 85, 10.1016/j.molmet.2024.101962, 101962. 38815625 PMC11180345

[17] Min B. H. , Devi S. , and Kwon G. H. , et al.Gut Microbiota-Derived Indole Compounds Attenuate Metabolic Dysfunction-Associated Steatotic Liver Disease by Improving Fat Metabolism and Inflammation, Gut Microbes. (2024) 16, no. 1, 10.1080/19490976.2024.2307568, 2307568. 38299316 PMC10841017

[18] Pentkowski N. S. , Rogge-Obando K. K. , Donaldson T. N. , Bouquin S. J. , and Clark B. J. , Anxiety and Alzheimer’s Disease: Behavioral Analysis and Neural Basis in Rodent Models of Alzheimer’s-Related Neuropathology, Neuroscience & Biobehavioral Reviews. (2021) 127, 647–658, 10.1016/j.neubiorev.2021.05.005. 33979573 PMC8292229

[19] Siddle M. , Gallego Durán R. D. , Goel D. , Renquist B. J. , Holt M. K. , and Hadjihambi A. , Mechanistic Insights Into the Liver-Brain Axis During Chronic Liver Disease, Nature Reviews Gastroenterology & Hepatology. (2025) 1–23, 10.1038/s41575-025-01142-z. PMC13034676 41214287

[20] Kjærgaard K. , Daugaard Mikkelsen A. C. , and Landau A. M. , et al.Cognitive Dysfunction in Early Experimental Metabolic Dysfunction-Associated Steatotic Liver Disease Is Associated With Systemic Inflammation and Neuroinflammation, JHEP Reports. (2024) 6, no. 3, 10.1016/j.jhepr.2023.100992, 100992. 38415019 PMC10897893

[21] Han H. , Ge X. , and Komakula S. S. B. , et al.Macrophage-Derived Osteopontin (SPP1) Protects From Nonalcoholic Steatohepatitis, Gastroenterology. (2023) 165, no. 1, 201–217, 10.1053/j.gastro.2023.03.228. 37028770 PMC10986640

[22] Bisgaard T. H. , Allin K. H. , Keefer L. , Ananthakrishnan A. N. , and Jess T. , Depression and Anxiety in Inflammatory Bowel Disease: Epidemiology, Mechanisms and Treatment, Nature Reviews Gastroenterology & Hepatology. (2022) 19, no. 11, 717–726, 10.1038/s41575-022-00634-6. 35732730

[23] Zhang S. , Gao Y. , Zhao Y. , Huang T. Y. , Zheng Q. , and Wang X. , Peripheral and Central Neuroimmune Mechanisms in Alzheimer’s Disease Pathogenesis, Molecular Neurodegeneration. (2025) 20, no. 1, 10.1186/s13024-025-00812-5. PMC11846304 39985073

[24] Liu J. H. , Zhang M. , and Wang Q. , et al.Distinct Roles of Astroglia and Neurons in Synaptic Plasticity and Memory, Molecular Psychiatry. (2022) 27, no. 2, 873–885, 10.1038/s41380-021-01332-6. 34642458

[25] Varma V. R. , Desai R. J. , and Navakkode S. , et al.Hydroxychloroquine Lowers Alzheimer’s Disease and Related Dementias Risk and Rescues Molecular Phenotypes Related to Alzheimer’s Disease, Molecular Psychiatry. (2023) 28, no. 3, 1312–1326, 10.1038/s41380-022-01912-0. 36577843 PMC10005941

[26] Kozachkov L. , Kastanenka K. V. , and Krotov D. , Building Transformers From Neurons and Astrocytes, Proceedings of the National Academy of Sciences. (2023) 120, no. 34, 10.1073/pnas.2219150120. PMC10450673 37579149

[27] González-Arias C. , Sánchez-Ruiz A. , and Esparza J. , et al.Dysfunctional Serotonergic Neuron-Astrocyte Signaling in Depressive-Like States, Molecular Psychiatry. (2023) 28, no. 9, 3856–3873, 10.1038/s41380-023-02269-8. 37773446 PMC10730416

[28] Yang L. , Youngblood H. , Wu C. , and Zhang Q. , Mitochondria as a Target for Neuroprotection: Role of Methylene Blue and Photobiomodulation, Translational Neurodegeneration. (2020) 9, no. 1, 10.1186/s40035-020-00197-z. PMC7262767 32475349

[29] Verma A. , Shteinfer-Kuzmine A. , and Kamenetsky N. , et al.Targeting the Overexpressed Mitochondrial Protein VDAC1 in a Mouse Model of Alzheimer’s Disease Protects Against Mitochondrial Dysfunction and Mitigates Brain Pathology, Translational Neurodegeneration. (2022) 11, no. 1, 10.1186/s40035-022-00329-7. PMC9795455 36578022

[30] Skovira J. W. , Wu J. , and Matyas J. J. , et al.Cell Cycle Inhibition Reduces Inflammatory Responses, Neuronal Loss, and Cognitive Deficits Induced by Hypobaria Exposure Following Traumatic Brain Injury, Journal of Neuroinflammation. (2016) 13, no. 1, 10.1186/s12974-016-0769-2, 2-s2.0-85000360945. PMC5131508 27903275

[31] Vallianou N. , Christodoulatos G. S. , and Karampela I. , et al.Understanding the Role of the Gut Microbiome and Microbial Metabolites in Non-Alcoholic Fatty Liver Disease: Current Evidence and Perspectives, Biomolecules. (2022) 12, no. 1, 10.3390/biom12010056. PMC8774162 35053205

[32] Aghara H. , Patel M. , Chadha P. , Parwani K. , Chaturvedi R. , and Mandal P. , Unraveling the Gut-Liver-Brain Axis: Microbiome, Inflammation, and Emerging Therapeutic Approaches, Mediators of Inflammation. (2025) 2025, no. 1, 10.1155/mi/6733477, 6733477. 40568349 PMC12197523

[33] Haase S. , Wilck N. , Haghikia A. , Gold R. , Mueller D. N. , and Linker R. A. , The Role of the Gut Microbiota and Microbial Metabolites in Neuroinflammation, European Journal of Immunology. (2020) 50, no. 12, 1863–1870, 10.1002/eji.201847807. 33188704

[34] Zhang J. , Chen K. , and Chen F. , Exploring the Impact of the Liver-Intestine-Brain Axis on Brain Function in Non-Alcoholic Fatty Liver Disease, Journal of Pharmaceutical Analysis. (2025) 15, no. 5, 10.1016/j.jpha.2024.101077, 101077. 40433559 PMC12104701

[35] Liu D. , Nguyen T. T. L. , and Gao H. , et al.TCF7L2 lncRNA: A Link Between Bipolar Disorder and Body Mass Index through Glucocorticoid Signaling, Molecular Psychiatry. (2021) 26, no. 12, 7454–7464, 10.1038/s41380-021-01274-z. 34535768 PMC8872993

[36] Lee D. S. , An T. H. , and Kim H. , et al.Tcf7l2 in Hepatocytes Regulates de Novo Lipogenesis in Diet-Induced Non-Alcoholic Fatty Liver Disease in Mice, Diabetologia. (2023) 66, no. 5, 931–954, 10.1007/s00125-023-05878-8. 36759348 PMC10036287

[37] Wang L. , Zhang L. , and Wang K. , et al.Microglial Lcn2 Knockout Enhances Chronic Intracerebral Hemorrhage Recovery by Restoring Myelin and Reducing Inflammation, Theranostics. (2025) 15, no. 10, 4763–4784, 10.7150/thno.109440. 40225581 PMC11984404

[38] Jung B. K. , Park Y. , and Yoon B. , et al.Reduced Secretion of LCN2 (lipocalin 2) From Reactive Astrocytes Through Autophagic and Proteasomal Regulation Alleviates Inflammatory Stress and Neuronal Damage, Autophagy. (2023) 19, no. 8, 2296–2317, 10.1080/15548627.2023.2180202. 36781380 PMC10351455

[39] Huang Q. , Xing J. , and Li G. , et al.LCN2 Regulates the Gut Microbiota and Metabolic Profile in Mice Infected With *Mycobacterium bovis* , MSystems. (2024) 9, no. 8, 10.1128/msystems.00501-24. PMC11334432 39051782

[40] Thanos J. M. , Campbell O. C. , and Cowan M. N. , et al.STING Deletion Protects Against Amyloid β-Induced Alzheimer’s Disease Pathogenesis, Alzheimers & Dementia. (2025) 21, no. 5, 10.1002/alz.70305. PMC12101966 40410932

[41] Sadanandan J. , Sathyanesan M. , and Newton S. S. , Aging Alters the Expression of Trophic Factors and Tight Junction Proteins in the Mouse Choroid Plexus, Fluids and Barriers of the CNS. (2024) 21, no. 1, 2024–2077, 10.1186/s12987-024-00574-0. PMC11438291 39334352

[42] Mai W. , Peng D. , and Wu L. , et al.Maternal Cadmium Exposure Promotes Amyloid-Beta Pathology by Interrupting the Interaction of AQP1 and HCN1 in Male Offspring, Ecotoxicology and Environmental Safety. (2025) 305, 10.1016/j.ecoenv.2025.119238, 119238. 41125051

[43] Eefsen M. , Jelnes P. , Schmidt L. E. , Vainer B. , Bisgaard H. C. , and Larsen F. S. , Brain Expression of the Water Channels Aquaporin-1 and -4 in Mice With Acute Liver Injury, Hyperammonemia and Brain Edema, Metabolic Brain Disease. (2010) 25, no. 3, 315–323, 10.1007/s11011-010-9213-y, 2-s2.0-78149406936. 20938728

[44] Bo T. , Gao L. , and Yao Z. , et al.Hepatic Selective Insulin Resistance at the Intersection of Insulin Signaling and Metabolic Dysfunction-Associated Steatotic Liver Disease, Cell Metabolism. (2024) 36, no. 5, 947–968, 10.1016/j.cmet.2024.04.006. 38718757

[45] Bansal S. K. and Bansal M. B. , Pathogenesis of MASLD and MASH-Role of Insulin Resistance and Lipotoxicity, Alimentary Pharmacology & Therapeutics. (2024) 59, no. S1, S10–S22, 10.1111/apt.17930. 38451123

[46] Li Y. , Yang P. , Ye J. , Xu Q. , Wu J. , and Wang Y. , Updated Mechanisms of MASLD Pathogenesis, Lipids in Health and Disease. (2024) 23, no. 1, 2024–2117, 10.1186/s12944-024-02108-x. PMC11034170 38649999

[47] Medina-Julio D. , Ramírez-Mejía M. M. , Cordova-Gallardo J. , Peniche-Luna E. , Cantú-Brito C. , and Mendez-Sanchez N. , From Liver to Brain: How MAFLD/MASLD Impacts Cognitive Function, Medical Science Monitor: International Medical Journal of Experimental and Clinical Research. (2024) 30, 10.12659/MSM.943417. PMC10836032 38282346

[48] Lee H. G. , Lee J. H. , Flausino L. E. , and Quintana F. J. , Neuroinflammation: An Astrocyte Perspective, Science Translational Medicine. (2023) 15, no. 721, 10.1126/scitranslmed.adi7828. 37939162

