# Supplementary material for: Inflammatory Factors Derived From Metabolic Dysfunction‐Alcoholic Fatty Liver Disease: Inducers of Anxiety and Spatial Memory Impairment
Source: Mediators Inflamm. 2026 Feb 9;2026:5555342. doi: 10.1155/mi/5555342 (PMC12887228; doi:10.1155/mi/5555342)
Supplement: Supplementary file 1 — Supporting Information 1 Figure S1: The scatter plot of MR results. The more consistent in direction and the smaller the dispersion among the five methods, the greater the consistency in MR results. Figure S2: The single‐SNP plots of MR results. It represents the individual MR analysis results for each SNP. Figure S3: The funnel plots of MR results. Figure S4: The leave‐one‐out forest plots of MR results. [file MI-2026-5555342-s002.docx]

**Supplementary Method**

**Mendelian Randomization (MR)**

Mendelian Randomization (MR) is an epidemiological methodology that employs genetic variants as instrumental variables (IVs) to infer causal relationships between exposure factors (e.g., Metabolic Dysfunction-Associated Steatotic Liver Disease [MASLD]) and health outcomes (e.g., cognitive function). Its theoretical foundation derives from Mendel's Law of Independent Assortment, which states that allele transmission from parents to offspring follows a random pattern analogous to randomized controlled trial (RCT) mechanisms, thereby minimizing interference from postnatal environmental confounders[1].

In our proposed study investigating the potential causal effect of MASLD on cognitive impairment, conventional RCT approaches face limitations due to residual confounding (e.g., dietary patterns, aging). MR analysis addresses this through three methodological pillars:

(1) IV Selection: Identification of single-nucleotide polymorphisms (SNPs) with strong genome-wide association (p<5×10^⁻6^) to MASLD.

(2) Genetic Randomization: Populations bearing these MASLD-associated SNPs exhibit intrinsically elevated disease susceptibility regardless of lifestyle factors.

(3) Causal Inference: A positive association between these genetic proxies for MASLD risk and cognitive function incidence would substantiate causal effects, thereby eliminating confounding bias and reverse causation – a methodological strength inherent to MR designs.

**Study design**

This study design adhered to the guidelines outlined in STROBE-MR (Strengthening the Reporting of Observational Studies in Epidemiology using Mendelian Randomization) (Supplementary Table 1). The causal inferences drawn from the estimates were based on three key assumptions[2]:

(1) Relevance assumption: The single-nucleotide polymorphisms (SNPs) used as instrumental variables were robustly associated with the exposure of interest.

(2) Independence assumption: The SNPs employed as instrumental variables were not associated with any potential confounders.

(3) Exclusion restriction assumption: The SNPs exhibited associations with the outcome solely through their impact on the exposure, without any alternative pathways.

These assumptions formed the foundation for the causal interpretation of the estimated associations.

**Data sources**

The characteristics of the GWAS data sources used in this study were summarized in Supplementary Table 2.

For MASLD, we obtained publicly aggregated data from the Finnish R12 database (<https://www.finngen.fi/fi>). This database contained genetic data related to the electronic health records of 500,000 Finns and included 13504 MASLD cases and 496844 controls.

For cognitive function, we selected eight outcome indicators which are all European ancestry individuals from the GWAS Catalog:

1. Cognitive processing speed (GCST90446168, n=2,266,733)
2. Cognitive processing accuracy (GCST90446169, n=1,783,727)
3. Word reading (GCST90104463, n=17,278)
4. Non-word reading (GCST90104465, n=16,746)
5. Spelling (GCST90104467, n=17,278)
6. Phoneme awareness (GCST90104469, n=12,411)
7. Worrier/anxious feelings (GCST90041879, n=1,044,404)
8. Depression (GCST90275155, n=63,875).

**Data extraction**

To fulfill the requirements of MR analysis, we followed a step-by-step process for data selection. Firstly, we selected SNPs associated with MASLD with a P-value of less than 5×10-6. We then applied the clump_data function in the 'TwoSampleMR' R package (R2＜0.001，kb=10000) to remove any unbalanced aggregation for each exposure[3]. We included SNPs with an effect allele frequency greater than 0.01 to avoid weak instrumental bias and excluded SNPs with an F-statistic less than <10[4]. The variance (R2) and instrumental strength (F-statistic) were calculated according to the following equation：

R2 = (2β2×MAF×(1-MAF))/(2β2×MAF×(1-MAF) + 2 N × MAF × (1-MAF) ×SE2)

F = R2(n−k−1)/k(1−R2) (where MAF = effect allele frequency, β = effect estimate of the SNP in the exposure GWAS, SE = standard error, N = sample size). Then we collated information about all phenotypes strongly associated with exposure SNPs on LD-link; SNPs strongly associated with confounding factors were excluded. The MR-Presso method was applied to test for heterogeneity and identify outlier SNPs, which were subsequently excluded from the analysis. Finally, we extracted and integrated the exposed SNP information to synergize it with the resultant data, so that the effect value of the resultant effect allele was consistent with that of the exposed one, and excluded the SNPs of the palindromes, finally obtaining the data used for the formal analysis of MR.

**MR analyses to elucidate causality**

We conducted a two-sample MR analysis to investigate the causal relationship between MASLD and Cognitive function. Effect estimates were obtained using the inverse variance weighting (IVW) method, which was a traditional approach in MR analysis. The results were reported as beta (β) values and standard errors for continuous outcomes and as ratios of ratios (ORs) and 95% confidence intervals (CIs) for binary outcomes. A significance level of P< 0.05 was considered nominally significant. IVW was used as the main method of MR analysis to ensure reliable conclusions. IVW was a method that meta-aggregates the effects of multiple loci when analyzing multiple SNPs[5].

**Sensitivity analyses**

As sensitivity analyses, four additional MR methods, namely MR Egger, Weighted median, Simple mode, and Weighted mode were employed to generate effect estimates, considering different assumptions regarding pleiotropy. These methods provided additional insights into the robustness of the results[6].

The presence of horizontal polytropy was tested by calculating the MR-Egger intercept[7]. A significance level of P<0.05 for the MR-Egger intercept indicated the presence of horizontal pleiotropy. Forest plots and funnel plots were used to visualize the results of the horizontal pleiotropy test. Heterogeneity was assessed using Cochrane’s Q test, with a significance level of P<0.05 indicating the presence of heterogeneity among the studies.

To assess the stability of the Mendelian randomization effect estimates and identify any significant outliers, a "leave-one-out" sensitivity analysis was performed. This analysis involved systematically removing each SNP from the analysis one at a time and re-performing the MR analysis. If any SNPs were found to have a substantial impact on the overall effect estimates, they would be removed, and the MR analysis would be repeated.

All main analysis methods and sensitivity tests were conducted using the ‘TwoSampleMR’, ‘tidyverse’, and ‘ggplot2’ packages in R software version 4.3.2. These packages provided the necessary functions for performing MR analyses and for data visualization.

**Supplementary Results**

**Genetic causality and correlation between MASLD and cognitive function**

Main analyses: The MASLD analysis in the Finnish R12 database utilized 21 SNPs. Some special SNPs were excluded because of palindromes or failure to pass the leave-one-out method (Supplementary Table 3). An F-statistic greater than 10 was considered informative for MR analyses, ensuring the validity of the instruments. When evaluating the causal efforts of MASLD and cognitive function using the IVW method, we found that MASLD has a strong genetic association with all five outcomes (Figure 2). The Scatterplot visualizing the results of the main MR analysis is presented in Supplementary Figure 1. MASLD might lead to a high risk of a decline in Cognitive processing accuracy (OR 0.995, 95% CI 0.991-0.999; P=0.027), Non-word reading (OR 0.958, 95% CI 0.926-0.991; P=0.015), Spelling (OR 0.966, 95% CI 0.933-0.999; P=0.045), and Phoneme awareness (OR 0.943, 95% CI 0.898-0.991; P=0.022). And MASLD might lead to a high risk of an increase in anxious feelings (OR 1.015, 95% CI 1.002-1.028; P=0.022). The results of four other methods can be found in Supplementary Table 4.

Sensitivity analyses: Cochran's Q-test was performed to assess heterogeneity in the IVW and MR-Egger regression results, and no significant heterogeneity was observed. Horizontal pleiotropy tests using MR-Egger showed that the Egger_intercept was not statistically significant, indicating no evidence of horizontal pleiotropic (Supplementary Table 5). Single-SNP plots and Funnel plots and leave-one-out forest plots demonstrated no significant effects from individual SNPs on the overall results (Supplementary Figure 2-4). These sensitivity analyses supported the robustness of the results.

**References**

1. Ference, B. A.; Holmes, M. V.; Smith, G. D., Using Mendelian Randomization to Improve the Design of Randomized Trials. *Cold Spring Harb Perspect Med* **2021,** 11, (7).

2. Skrivankova, V. W.; Richmond, R. C.; Woolf, B. A. R.; Yarmolinsky, J.; Davies, N. M.; Swanson, S. A.; VanderWeele, T. J.; Higgins, J. P. T.; Timpson, N. J.; Dimou, N.; Langenberg, C.; Golub, R. M.; Loder, E. W.; Gallo, V.; Tybjaerg-Hansen, A.; Davey Smith, G.; Egger, M.; Richards, J. B., Strengthening the Reporting of Observational Studies in Epidemiology Using Mendelian Randomization: The STROBE-MR Statement. *Jama* **2021,** 326, (16), 1614-1621.

3. Sudmant, P. H.; Rausch, T.; Gardner, E. J.; Handsaker, R. E.; Abyzov, A.; Huddleston, J.; Zhang, Y.; Ye, K.; Jun, G.; Fritz, M. H.; Konkel, M. K.; Malhotra, A.; Stütz, A. M.; Shi, X.; Casale, F. P.; Chen, J.; Hormozdiari, F.; Dayama, G.; Chen, K.; Malig, M.; Chaisson, M. J. P.; Walter, K.; Meiers, S.; Kashin, S.; Garrison, E.; Auton, A.; Lam, H. Y. K.; Mu, X. J.; Alkan, C.; Antaki, D.; Bae, T.; Cerveira, E.; Chines, P.; Chong, Z.; Clarke, L.; Dal, E.; Ding, L.; Emery, S.; Fan, X.; Gujral, M.; Kahveci, F.; Kidd, J. M.; Kong, Y.; Lameijer, E. W.; McCarthy, S.; Flicek, P.; Gibbs, R. A.; Marth, G.; Mason, C. E.; Menelaou, A.; Muzny, D. M.; Nelson, B. J.; Noor, A.; Parrish, N. F.; Pendleton, M.; Quitadamo, A.; Raeder, B.; Schadt, E. E.; Romanovitch, M.; Schlattl, A.; Sebra, R.; Shabalin, A. A.; Untergasser, A.; Walker, J. A.; Wang, M.; Yu, F.; Zhang, C.; Zhang, J.; Zheng-Bradley, X.; Zhou, W.; Zichner, T.; Sebat, J.; Batzer, M. A.; McCarroll, S. A.; Mills, R. E.; Gerstein, M. B.; Bashir, A.; Stegle, O.; Devine, S. E.; Lee, C.; Eichler, E. E.; Korbel, J. O., An integrated map of structural variation in 2,504 human genomes. *Nature* **2015,** 526, (7571), 75-81.

4. Burgess, S.; Thompson, S. G., Avoiding bias from weak instruments in Mendelian randomization studies. *Int J Epidemiol* **2011,** 40, (3), 755-64.

5. Burgess, S.; Scott, R. A.; Timpson, N. J.; Davey Smith, G.; Thompson, S. G., Using published data in Mendelian randomization: a blueprint for efficient identification of causal risk factors. *Eur J Epidemiol* **2015,** 30, (7), 543-52.

6. Bowden, J.; Davey Smith, G.; Burgess, S., Mendelian randomization with invalid instruments: effect estimation and bias detection through Egger regression. *Int J Epidemiol* **2015,** 44, (2), 512-25.

7. Hemani, G.; Zheng, J.; Elsworth, B.; Wade, K. H.; Haberland, V.; Baird, D.; Laurin, C.; Burgess, S.; Bowden, J.; Langdon, R.; Tan, V. Y.; Yarmolinsky, J.; Shihab, H. A.; Timpson, N. J.; Evans, D. M.; Relton, C.; Martin, R. M.; Davey Smith, G.; Gaunt, T. R.; Haycock, P. C., The MR-Base platform supports systematic causal inference across the human phenome. *Elife* **2018,** 7.


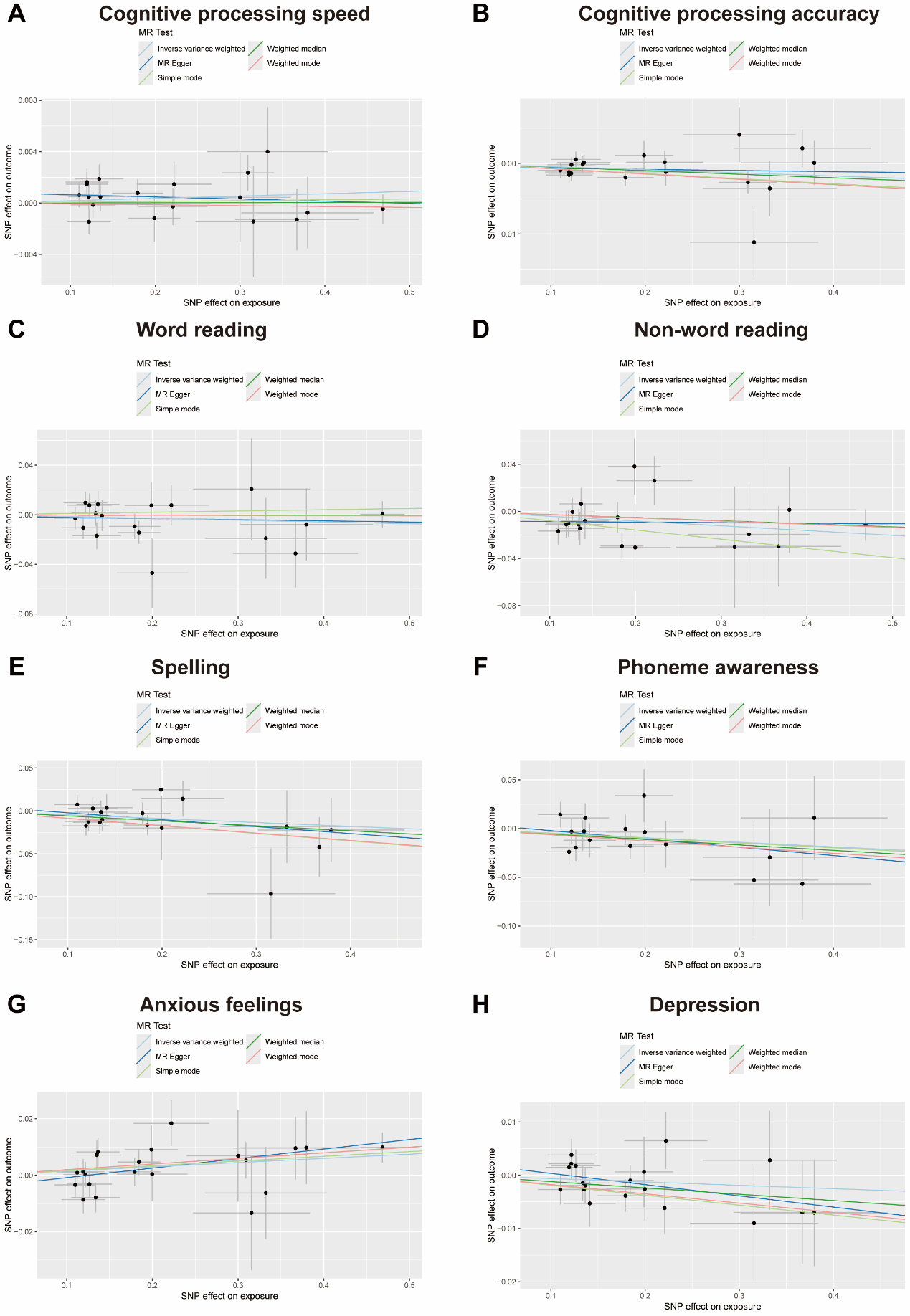


**Supplementary Figure 1. The Scatterplots display the results of MR with MASLD as the exposure and eight different outcomes.** (A-H) show the MR of MASLD as the exposure in relation to eight different outcomes, respectively. Each panel represents an independent MR analysis, using the IVW method as the primary analysis approach. (A) Cognitive processing speed. (B) Cognitive processing accuracy. (C) Word reading. (D) Non-word reading. (E) Spelling. (F) Phoneme awareness. (G) anxious feelings. (H) Depression. The more consistent in direction and the smaller the dispersion among the five methods, the greater the consistency in MR results.


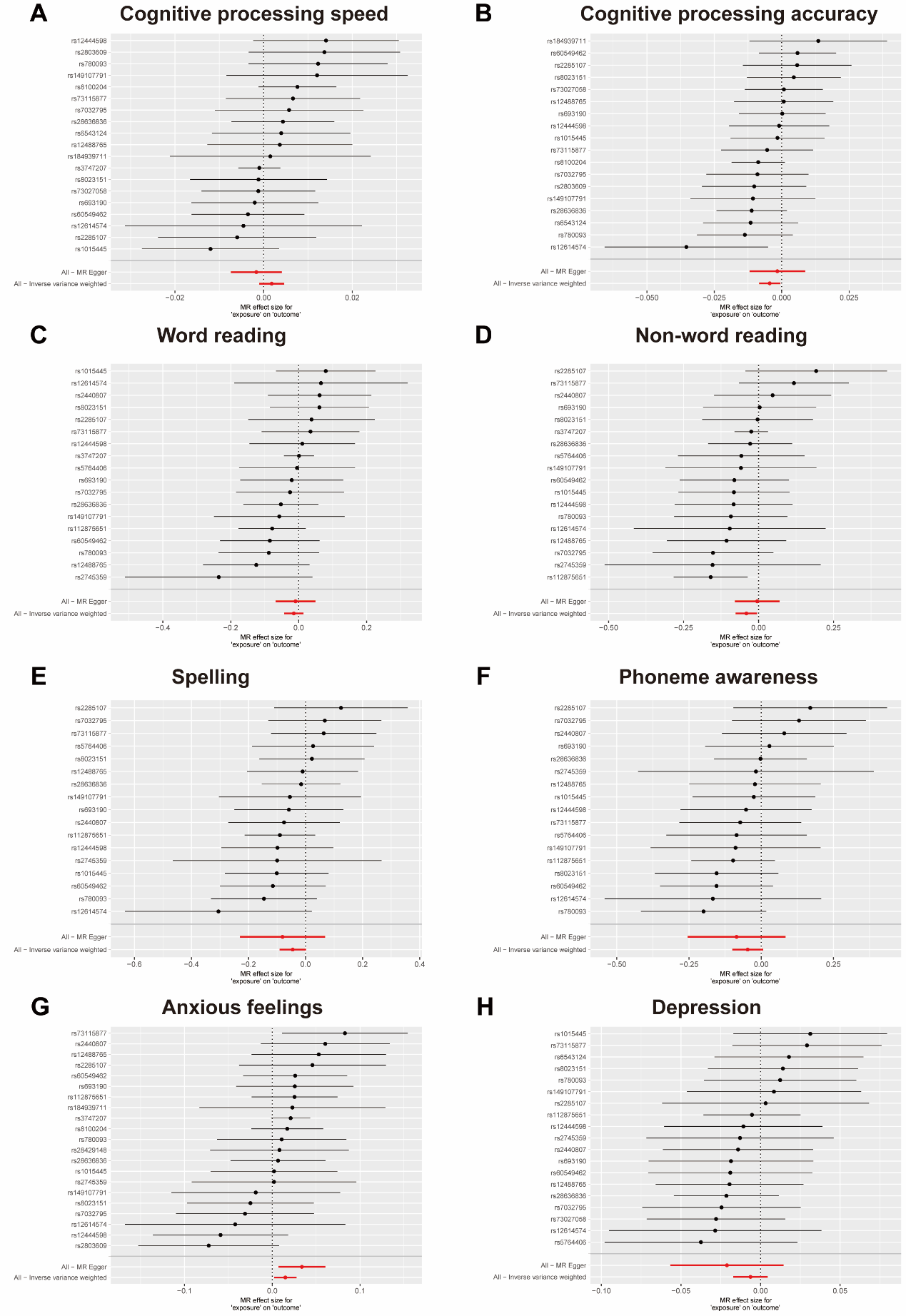


**Supplementary Figure 2. Single-SNP MR analyses of MASLD on eight outcomes.** (A-H) present MR evaluating the causal relationship between MASLD and eight different outcomes using the single-SNP approach. Each panel displays the causal effect estimate for each individual genetic instrument utilized in the analysis. (A) Cognitive processing speed. (B) Cognitive processing accuracy. (C) Word reading. (D) Non-word reading. (E) Spelling. (F) Phoneme awareness. (G) anxious feelings. (H) Depression.


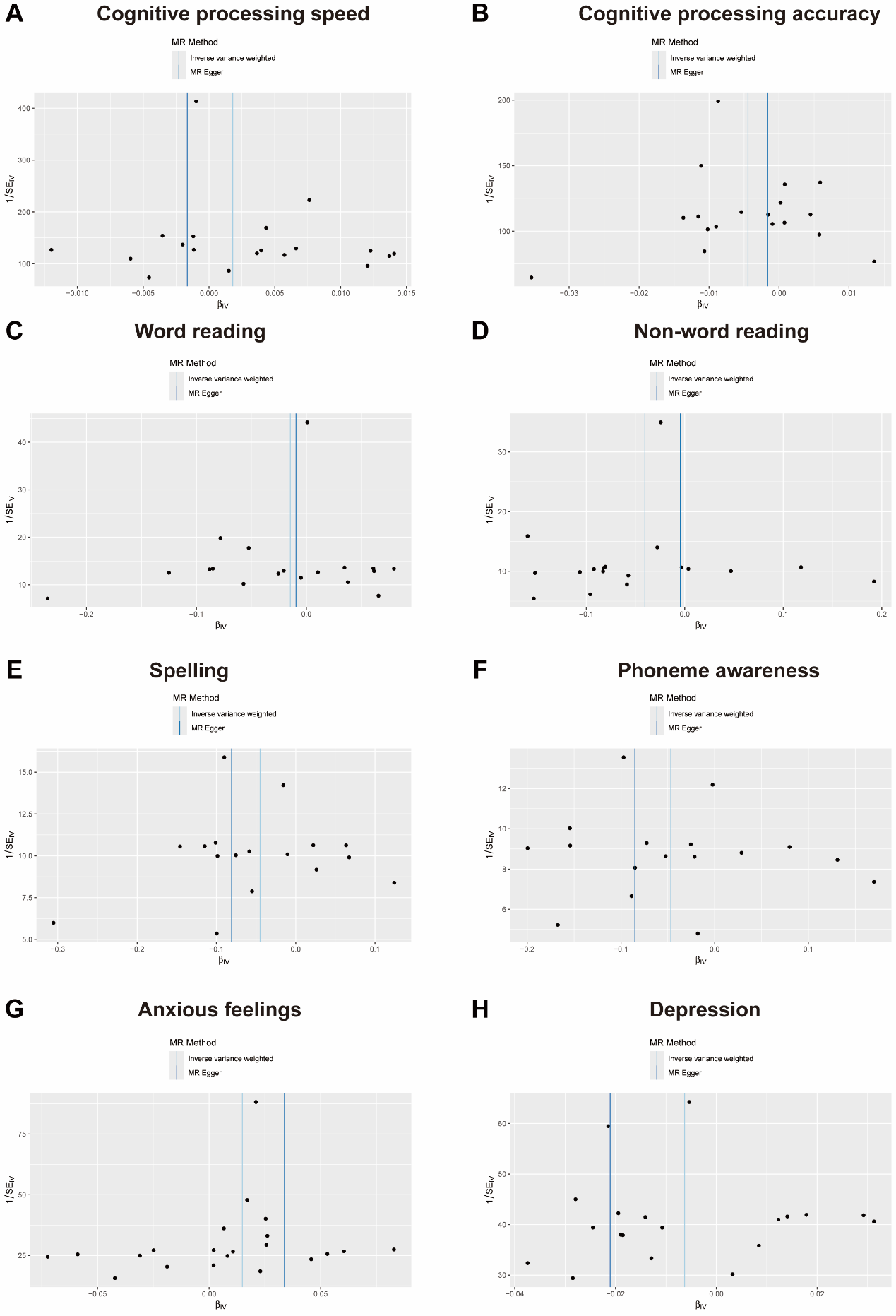


**Supplementary Figure 3. Funnel Plots for MR Analyses of MASLD on eight outcomes.** (A-H) systematically present funnel plots from MR examining MASLD as the exposure factor in relation to eight distinct outcomes. Each panel visually displays the relationship between effect estimates of all genetic instrumental variables and their precision, serving to evaluate the robustness of findings and identify potential biases. (A) Cognitive processing speed. (B) Cognitive processing accuracy. (C) Word reading. (D) Non-word reading. (E) Spelling. (F) Phoneme awareness. (G) anxious feelings. (H) Depression.


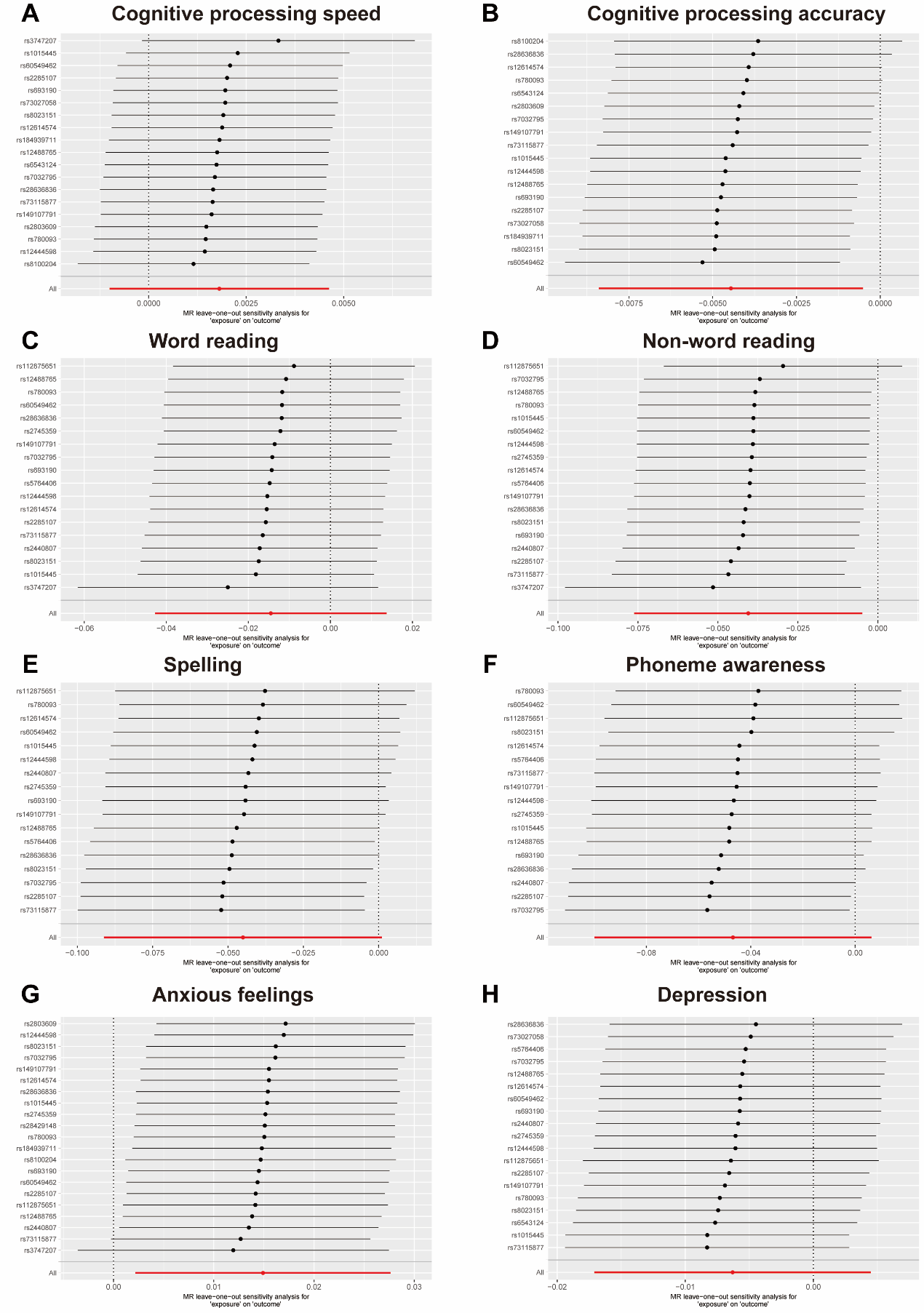


**Supplementary Figure 4. Leave-One-Out Sensitivity Analysis for MR of MASLD on Eight Outcomes.** (A-H) present leave-one-out sensitivity analyses from MR examining MASLD as exposure on eight outcomes. This figure illustrates the iterative process of excluding one SNP at a time from the dataset, performing MR analysis using the remaining SNP subset to derive an effect estimate without that particular SNP. This procedure is conducted for each SNP in the dataset consecutively. The ideal outcome requires that the post-exclusion estimates remain consistently clustered on the same side of the null line. (A) Cognitive processing speed. (B) Cognitive processing accuracy. (C) Word reading. (D) Non-word reading. (E) Spelling. (F) Phoneme awareness. (G) anxious feelings. (H) Depression.
